# Altered tumor microenvironment heterogeneity of penile cancer during progression from non‐lymphatic to lymphatic metastasis

**DOI:** 10.1002/cam4.70025

**Published:** 2024-07-14

**Authors:** Da‐Ming Xu, Xiao‐Yu Zhuang, Hua‐Li Ma, Zai‐Shang Li, Li‐Chao Wei, Jun‐Hang Luo, Hui Han

**Affiliations:** ^1^ State Key Laboratory of Oncology in South China, Guangdong Provincial Clinical Research Center for Cancer Sun Yat‐sen University Cancer Center Guangzhou P. R. China; ^2^ Department of Urology Sun Yat‐sen University Cancer Center Guangzhou P. R. China; ^3^ Department of Anesthesiology Second Affiliated Hospital of Shantou University Medical College Shantou P. R. China; ^4^ Department of Radiology Sun Yat‐sen University Cancer Center Guangzhou P. R. China; ^5^ Department of Urology, Shenzhen People's Hospital The Second Clinic Medical College of Jinan University Shenzhen P. R. China; ^6^ Department of Urology, First Affiliated Hospital Sun Yat‐sen University Guangzhou P. R. China; ^7^ Institute of Precision Medicine, First Affiliated Hospital, Sun Yat‐sen University Guangzhou P. R. China

**Keywords:** lymphatic metastasis, malignant progression, penile cancer, premetastatic niche, tumor microenvironment

## Abstract

**Background:**

Lymphatic metastasis is the major challenge in the treatment of penile cancer. The prognosis of individuals with lymphatic metastasis is extremely poor. Therefore, early identification of disease progression and lymphatic metastasis is an urgent task for researchers in penile cancer worldwide.

**Methods:**

In this study, using single‐cell RNA sequencing, an immune landscape was established for the cancer ecosystem based on 46,861 cells from six patients with penile cancer (four with lymphatic metastasis [stage IV] and two without lymphatic metastasis [stage I]). Using bulk RNA sequencing, the discrepancy between the cancers and their respective metastatic lymph nodes was depicted based on seven patients with penile cancer.

**Results:**

The interaction between epithelial cells, fibroblasts, and endothelial cells, and the functional cooperation among invasion, epithelial‐mesenchymal transition, and angiogenesis were found to be important landscapes in the penile cancer ecosystem, playing important roles in progression of cancer and lymph node metastasis.

**Conclusions:**

This study is the first to investigate the altered tumor microenvironment heterogeneity of penile cancer as it evolves from non‐lymphatic to lymphatic metastasis and provides insights into the mechanisms underlying malignant progression, the premetastatic niche, and lymphatic metastasis in penile cancer.

## 1 INTRODUCTION

In most solid cancers, lymphatic metastasis of the primary tumor precedes distant metastasis and portends a poor prognosis.^1^ Penile cancer is a very rare malignancy with a total incidence of 0.5–0.94 per 100,000 men in the European and American populations.^2^ The cognition limitation of penile cancer and the instability of the treatment effect are damaging to the physical and mental health of affected men and their families.^3^ Lymph node metastasis is the key determinant of the outcome in patients with penile cancer,^4^, ^5^ with N0, N1, N2, and N3 metastasis corresponding to respective 5‐year cancer‐specific survival rates of 95%, 80%, 65%, and 35%.^6^ Patients with penile cancer and pelvic lymph node or distant metastasis have been found to have a significantly lower 5‐year survival rate than those with only inguinal lymph node metastasis.^7^ Metastasis is not a single event, but rather a continuum of phenotypes.^8^, ^9^ Premetastatic features, namely, active metastasis‐related signals, such as epithelial‐mesenchymal transition (EMT) and angiogenic signaling, may already be present in the primary tumor before lymphatic metastasis. Occult metastases occur in 20%–25% of patients with penile cancer and N0 inguinal disease, and a nonrandomized, controlled trial has suggested that early lymph node dissection may benefit these patients.^10^ Therefore, early detection of lymphatic spread of penile cancer is crucial. However, current non‐invasive and invasive methods, including computed tomography and lymph node biopsy, cannot detect lymph node micrometastases with sufficient reliability.^3^, ^11^ Moreover, owing to the rarity of the disease, the mechanisms underlying progression and metastasis of penile cancer are still not well understood, which hinders progress in its treatment. Recently, immunotherapy has become an important treatment option for advanced metastatic penile cancer, but with variable response rates.^3^, ^12^, ^13^ With ongoing development of immunotherapy, there has been an increasing focus on the role of the tumor microenvironment in the metastatic cascade, especially with respect to lymphatic metastasis.^14^, ^15^, ^16^

We have speculated that the tumor microenvironment could shape the specificity and heterogeneity of penile cancer, induce lymphatic metastasis, and account for the different responses to immunotherapy. In this study, we investigated the tumor microenvironment in penile cancer. By analyzing single‐cell RNA sequencing and bulk RNA sequencing data from specimens of penile cancer at different stages and metastatic lymph nodes, we identified differences in the distribution of cancer‐associated immune cells and their contribution to disease progression. This research could lay the foundation for better clinical understanding of the progression, premetastatic niche and metastasis of penile cancer.

## 2 METHODS

### 2.1 Patients

A histological diagnosis of penile cancer was confirmed by specimens of the primary tumor or metastasis in lymph nodes. These specimens were pathologically confirmed as squamous cell carcinoma of the penis. Using single‐cell RNA sequencing, the immune landscape of the cancer ecosystem was established based on 46,861 cells from six patients with penile cancer (four with lymphatic metastasis [stage IV] and two without lymphatic metastasis [stage I]). Using bulk RNA sequencing, the discrepancy between the cancers and their respective metastatic lymph nodes was depicted based on another seven patients. None of the patients had received antineoplastic therapy before specimen collection. Age and HPV infection between the different groups has no statistical significance.

This research was approved by the Institutional Review Board of Sun Yat‐sen University Cancer Center (B2023‐390‐01) and conducted in accordance with the criteria set by the Declaration of Helsinki. The study design and conduct complied with all relevant regulations regarding the use of human study participants.

### 2.2 Single‐cell RNA sequencing experiments

#### 2.2.1 Control of cell quality

The same amount of 0.4% Trypan blue solution was added to appropriate volumes of single‐cell suspension. Cell counting was performed using a Countess® II Automated Cell Counter (Thermo Fisher Scientific, Waltham, MA, USA). The viable cell concentration was then adjusted to the ideal concentration for sequencing.

#### 2.2.2 Isolation of single cells using the 10× Genomics protocol

Gel Beads with the barcode information were allowed to combine with the cells and enzymes. A series of processes then generated Gel Beads in emulsion (GEMs). Sequences were captured, complementary DNA fragments were reverse‐transcribed, and the samples were labeled. The complementary DNA was used as a template for subsequent amplification by polymerase chain reaction. Finally, we were able to construct a standard sequencing library.

#### 2.2.3 Construction of a standard sequencing library

The complementary DNA was digested and broken down into 200–300‐bp fragments. After performing the conventional second‐generation sequencing library construction step, we obtained DNA libraries through amplification by polymerase chain reaction.

#### 2.2.4 Sequencing of libraries

We used PE150 mode in the Illumina sequencing platform to perform high‐throughput sequencing of the library.

#### 2.2.5 Further filtration of abnormal cells

After identification of gene expression using Cell Ranger software,^17^ the expression matrix was transferred to Seurat in R for analysis.^18^ First, GEMs including multiple cells were detected in each sample. DoubletFinder in R was used to calculate the probability of multicellularity in GEMs.^19^ Next, based on the relationship between the effective cell number according to the 10× official formulation (after filtering in Cell Ranger) and the multicellularity rate, we calculated the multicellularity rate in each sample, determined the multicellularity threshold in each sample, and performed multicellularity filtration.

#### 2.2.6 Classification of single‐cell subsets

After removing low‐quality cells, the data were merged and the batch effect was corrected using Harmony.^20^ Dimensionality reduction by principal component analysis was first performed on the combined data. We then calculated the global center of all datasets within each cluster, as well as the center of each specific dataset. Finally, within each cluster, the correction factor was calculated for each data set based on the center, and the cells were corrected to cluster towards the center. The above steps were repeated until the clustering effect became stable.

#### 2.2.7 Visualization of classification results

Based on the results of cell subset classification, UMAP was further applied to visualize the classification results for single‐cell subsets.

#### 2.2.8 Identification of single‐cell subsets

The above single‐cell subgroup classifications were clustered based on the similarity of cell expression features. SingleR was used for automated annotation of all cells.^21^

#### 2.2.9 Analysis of upregulated genes in clusters

The Wilcoxon rank‐sum test was used to compare the expression value of each gene in the target cluster with that of the rest of the cells. The screening criteria for a significantly upregulated gene were as follows: first, the genes was expressed in more than 25% of cells in target or control subsets; second, the *p*‐value was ≤0.01; third, gene expression was upregulated by ≥1.28‐fold.

### 2.3 Enrichment analysis

#### 2.3.1 Gene Ontology enrichment analysis

Gene Ontology (GO) is a comprehensive database that describes the functional attributes of genes in biological organisms.^22^ It consists of three ontologies describing molecular function, the cellular component, and the biological process.

#### 2.3.2 KEGG enrichment analysis

The KEGG database focuses on gene pathways within biological organisms.^23^ By determining significant enrichment through pathway analysis, we can identify major biochemical metabolic and signaling pathways involving differential genes.

#### 2.3.3 Disease Ontology enrichment analysis

The Disease Ontology (DO) database can be used to study the relationship between gene function and diseases.^24^

#### 2.3.4 Reactome enrichment analysis

Reactome is a specialized database that collects reaction and biological pathway associations for various species.^25^ We mapped the upregulated genes in each cell subpopulation to the terms in the GO, KEGG, DO, and Reactome databases and calculated the number of differentially expressed genes for each term, thereby obtaining a statistical count of differential genes associated with a specific GO, KEGG, DO, or Reactome function. We then applied a hypergeometric test to identify GO, KEGG, DO, and Reactome entries that were significantly enriched among the differential genes.

#### 2.3.5 Analysis of the cell cycle

We used the Seurat package in R to make a score assessment of the cell cycle for each cell based on the expression of marker genes containing G1 phase, S phase, G2 phase, and M phase.^26^ Cells with the highest score (<0.3) were defined as non‐cycle cells.^27^

#### 2.3.6 Analysis of cell status

The Seurat package in R and the CancerSEA database were used to assign a cell status score to each cell based on the expression level of the marker gene to explore the potential biological functions of each cell subpopulation.^28^

#### 2.3.7 Construction of single‐cell trajectories

Monocle (version 2.10.1) was used for single‐cell trajectory analysis based on gene expression levels, including differential gene analysis based on differentiation status, differential gene analysis based on pseudo‐time variation, and differential gene analysis based on different branches.^29^, ^30^

#### 2.3.8 Differential gene analysis between samples

The Seurat package in R and model‐based analysis of single‐cell transcriptomics were used to find differentially expressed genes for a group in one cluster.^18^

#### 2.3.9 Analysis of cell–cell communication

Cellchat software was used to analyze the communication between ligand‐receptor cells and their expression among cell clusters in a single‐cell gene expression matrix, which consisted of three aspects: prediction of communication of ligand‐receptor cells, cellular communication at the signaling path level, and analysis of the ligand‐receptor cell signaling path.^31^

#### 2.3.10 Bulk RNA sequencing experiments

Bulk RNA sequencing experiments were used to evaluate the differences in gene expression between penile cancers and their respective metastatic lymph nodes. These experiments consisted of a series of processes, including preparation of RNA samples, library preparation, data quality control, sequence alignment analysis, gene analysis, and expression level statistics.

## 3 RESULTS

### 3.1 Single‐cell RNA sequencing revealed the tumor microenvironment

**FIGURE 1:**
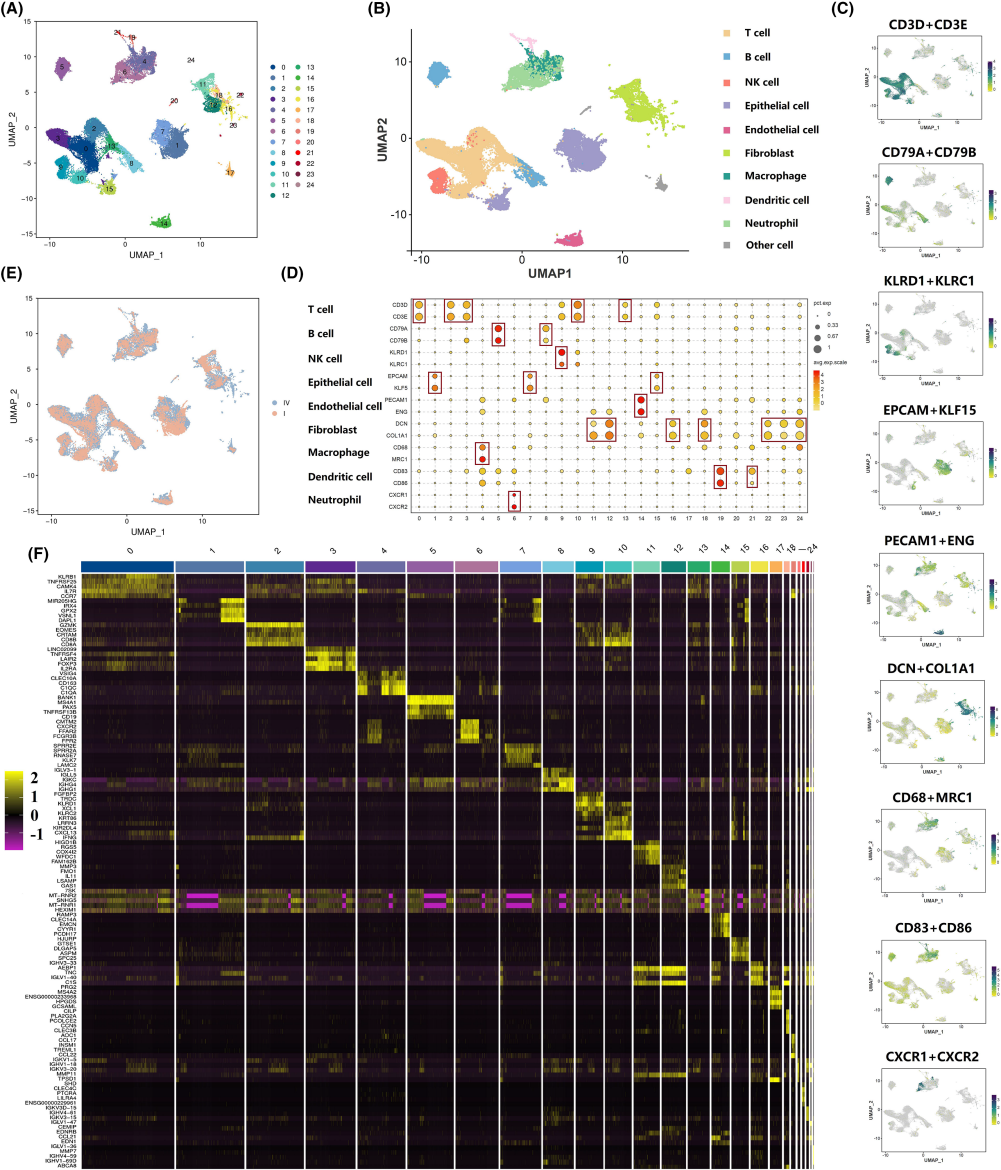
Distinct cellular constitutions in penile cancer delineated by single‐cell RNA sequencing analysis. (A) UMAP plots of 24 single‐cell clusters from all 46,861 cells separated by penile cancer; (B) nine main immune cell types in penile cancer microenvironment; (C, D) The main cell types annotated by known cell lineages in penile cancer; (E) The subgroup classification of all samples, containing stage IV group and stage I group; (F) Top 5 up‐regulation genes for each cluster.

A total of 46,861 cells were identified and classified into 24 separate clusters (Figure 1A), which consisted mainly of nine immune cell types, namely, T‐cell (*CD3D* and *CD3E*), B‐cell (*CD79A* and *CD79B*), natural killer cell (*KLRD1* and *KLRC1*), epithelial cell (*EPCAM* and *KLF5*), endothelial cell (ECs) (*PECAM1* and *ENG*), fibroblast (*DCN* and *COL1A1*), macrophage (*CD68* and *MRC1*), dendritic cell (*CD83* and *CD86*) and neutrophil (*CXCR1* and *CXCR2*) (Figure 1B–D). The distribution of groups (stage I and stage IV) was delineated using the UMAP (Uniform Manifold Approximation and Projection) algorithm (Figure 1E). We identified the genes that were upregulated for each of the 24 cell subpopulations as follows: cluster 0 (*n* = 634), cluster 1 (*n* = 1421), cluster 2 (*n* = 444), cluster 3 (*n* = 1007), cluster 4 (*n* = 1214), cluster 5 (*n* = 531), cluster 6 (*n* = 386), cluster 7 (*n* = 1353), cluster 8 (*n* = 180) cluster 9 (*n* = 580), cluster 10 (*n* = 873), cluster 11 (*n* = 1187), cluster 12 (*n* = 1746), cluster 13 (*n* = 199), cluster 14 (*n* = 718), cluster 15 (*n* = 2160), cluster 16 (*n* = 246), cluster 17 (*n* = 513), cluster 18 (*n* = 1460), cluster 19 (*n* = 1106), cluster 20 (*n* = 77), cluster 21 (*n* = 1487), cluster 22 (*n* = 197), cluster 23 (*n* = 200), and cluster 24 (*n* = 281). The top five differentially upregulated genes in each cluster were regarded as molecular characteristics (Figure 1F).

### 3.2 Malignant status of cells in the tumor microenvironment

**FIGURE 2:**
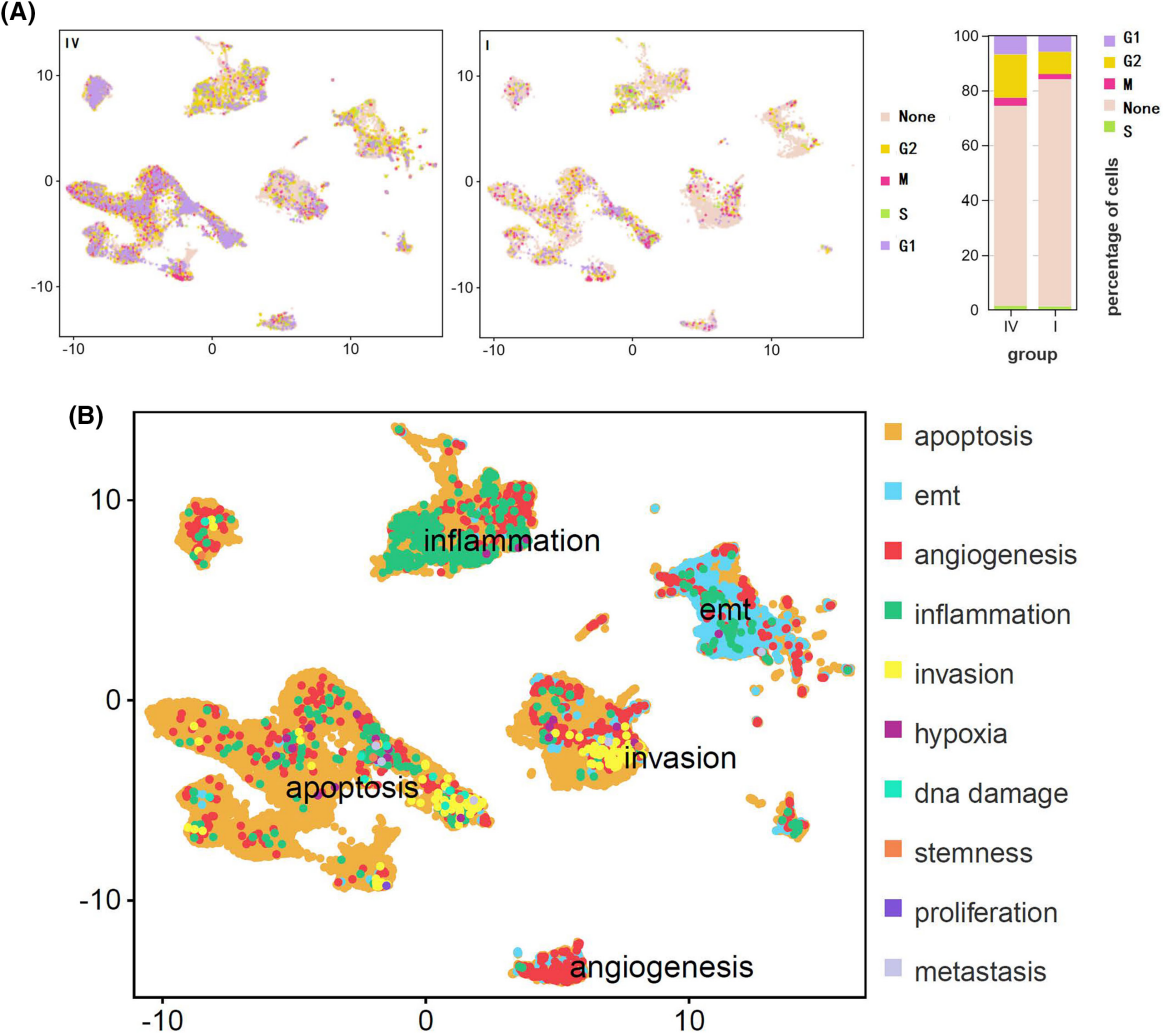
Cell cycle analysis, cell malignant status analysis and GSVA analysis in distinct cellular clusters. (A) Different cell cycle status between stage IV and stage I penile cancer; (B) cell malignant status analysis of distinct clusters, containing apoptosis, EMT, angiogenesis, inflammation, invasion, hypoxia, DNA damage, stemness, proliferation, and metastasis.

Next, we explored the malignant functional phenotypes of the cell clusters in lymphatic (stage IV) and non‐lymphatic (stage I) metastatic penile cancer in detail. As expected, there were more cells in the division stage in the stage IV group than in the stage I group (Figure 2A). As the tumors became more advanced, there were increases in the proportions of cells in G1 phase, S phase, G2 phase, and M phase of the cell cycle, suggesting increased activity of cell proliferation in stage IV disease. According to the CancerSEA database, the malignant functional cell phenotypes in penile cancer can be divided into five main states: apoptosis, inflammation, EMT, invasion, and angiogenesis (Figure 2B). Each cell cluster was used as the gene set for analysis of differences via the H item (hallmark gene sets) (Figure S1) and the C6 item (oncogenic signature gene sets) (Figure S2) in the MSigDB gene sets.

### 3.3 Cluster 1 demonstrated varying degrees of invasion in the single‐cell tumor landscape

**FIGURE 3:**
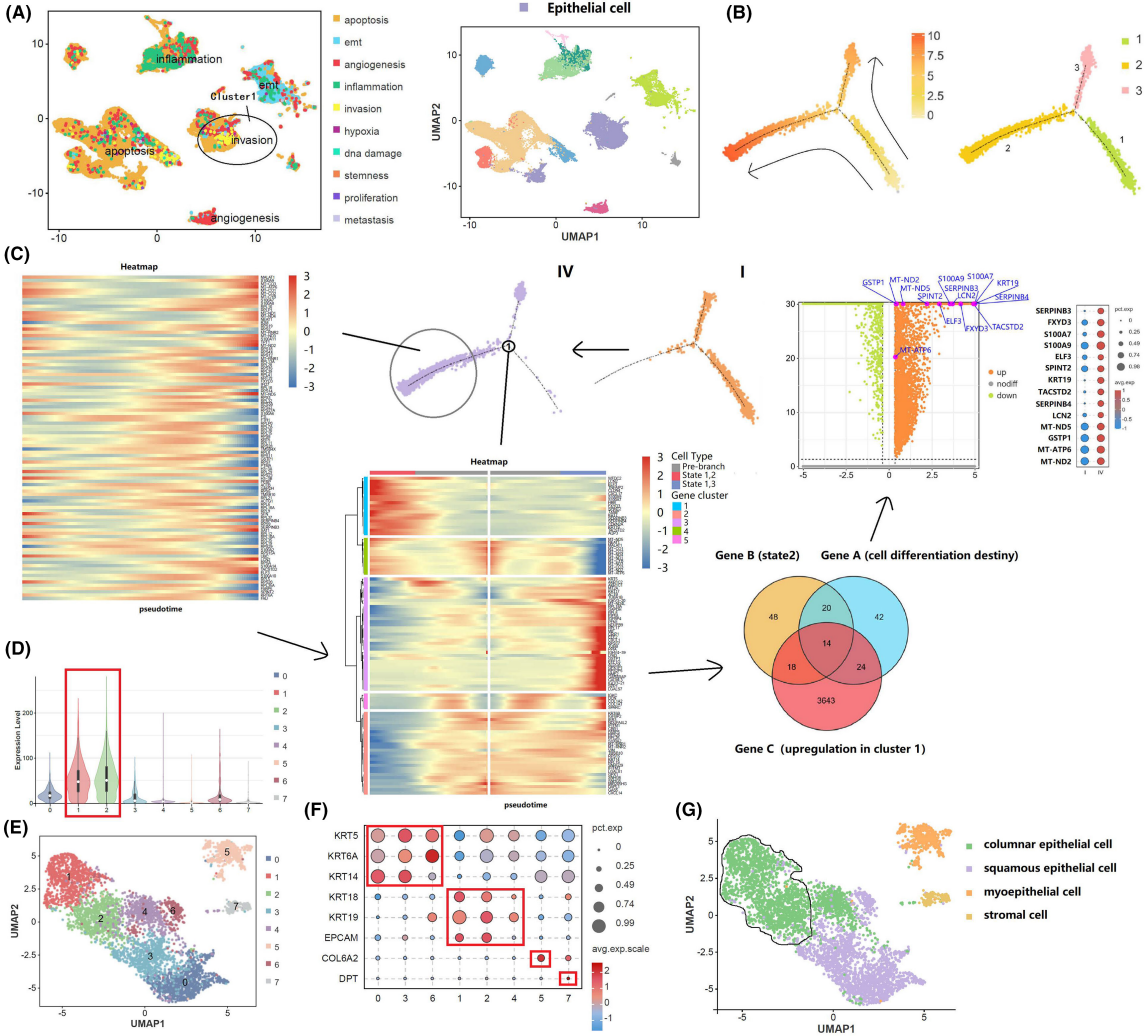
Cluster 1 demonstrated varying degrees of invasion in the single‐cell tumor landscape. (A) Cell malignant status analysis of cluster 1; (B) constructing single cell trajectories in cluster 1; (C) screening of major driver genes that dominate malignant behavior in cluster 1; (D) The overall expression intensity of target genes; (E) Single cell re‐clustering of cancer epithelial cell; (F, G) The cell type re‐annotation of cancer epithelial cell subsets.

We focused on cluster 1, which was composed mainly of epithelial cells that showed more enriched characteristics of invasiveness in comparison with the other subgroups (Figure 3A). We performed single‐cell trajectory and regrouping analyses in cluster 1 to identify potential differences in the tumor microenvironment between lymphatic and non‐lymphatic metastatic penile cancer. Pseudo‐time trajectory analysis of cell differentiation fate in cluster 1 identified three cell states (i.e., state 1, state 2, and state 3), which consisted of one root and two branches (Figure 3B). Comprehensive enrichment analyses of the upregulated gene set in cluster 1 were performed to clarify its role in various biological processes and tumor metabolic pathways (Figure S3). Notably, lymphatic metastatic penile cancer was more likely to select state 2 as a cell fate decision whereas non‐lymphatic metastatic penile cancer was more likely to select state 3, suggesting that state 2 was the direction for progression of malignancy. We identified the top 100 genes that could determine the cell differentiation fate, trajectory branch‐dependent genes for state 2, and significantly upregulated genes in cluster 1 and observed how they intersected in Venn diagrams. Next, a differential analysis of cells in cluster 1 was performed to confirm expression of the 14 marker genes (*SEPRINB3*, *FXYD3*, *S100A7*, *S100A9*, *ELF3*, *SPINT2*, *KRT19*, *TACSTD2*, *SERPINB4*, *LCN2*, *GSTP1*, *MT‐ND5*, *MT‐ATP6*, and *MT‐ND2*) obtained by analysis of the interactive Venn diagram (Figure 3C). Given that cluster 1 represents an invasive malignant state, it was reasonable to assume that the genes identified contribute to lymphatic metastasis by prompting epithelial cells to shape the invasion‐related microenvironment. Furthermore, the overall expression of 14 malignancy‐related target genes was analyzed (Figure 3D) and cell cluster 1 was grouped further (columnar epithelial cell [*KRT18*, *KRT19*, and *EPCAM*], squamous epithelial cell [*KRT5*, *KRT6A*, and *KRT14*], myoepithelial cell [*COL6A2*] and stromal cell [*DPT*]) based on previous research,^32^, ^33^ (Figure 3E–G). We found that the expression intensity of these 14 genes in columnar epithelial cell was higher than that in the other subgroups, which suggested that columnar epithelial cell plays a dominant role in malignant progression.

### 3.4 Cluster 12 showed varying degrees of EMT in the single‐cell tumor landscape

**FIGURE 4:**
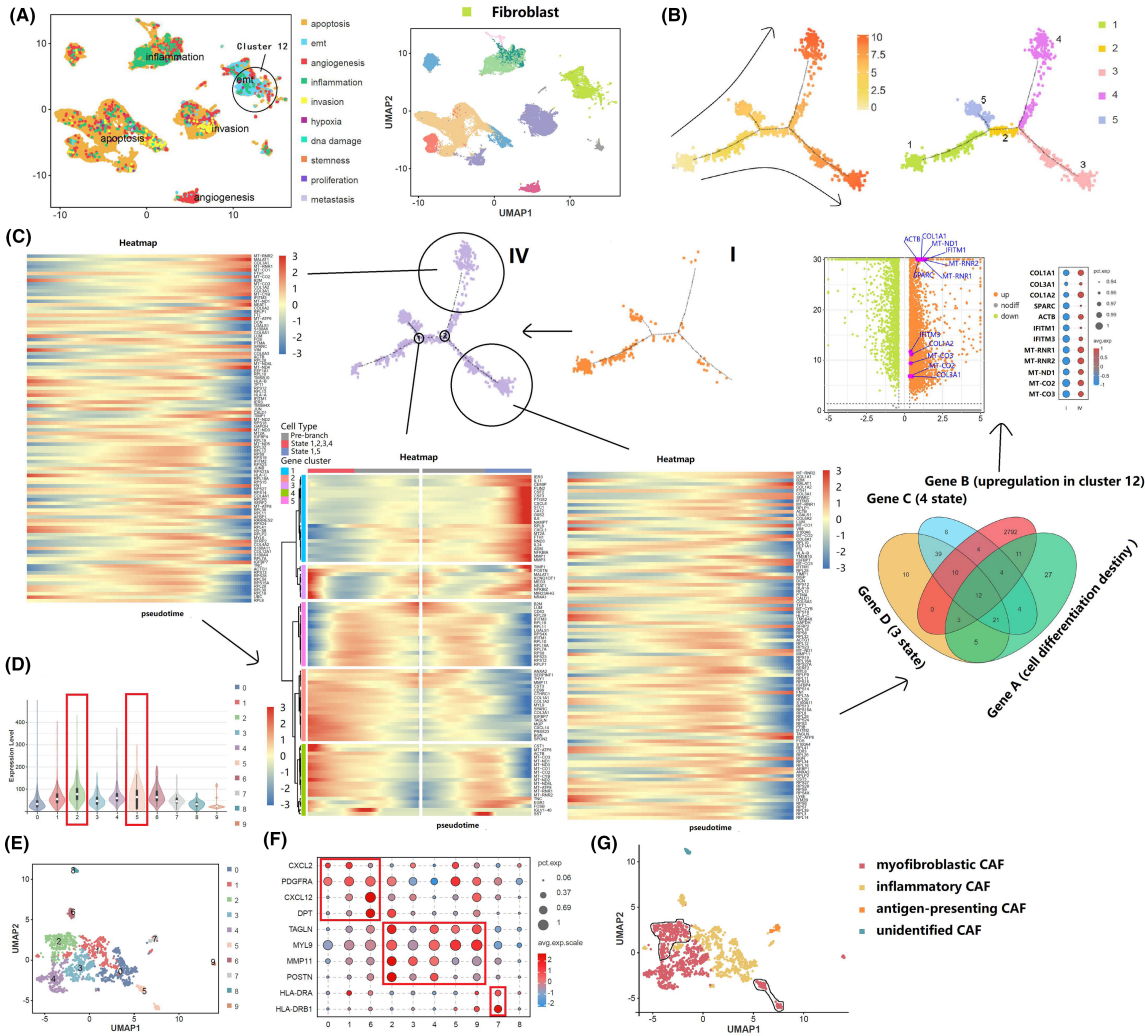
Cluster 12 showed varying degrees of EMT in the single‐cell tumor landscape. (A) Cell malignant status analysis of cluster 12; (B) constructing single cell trajectories in cluster 12; (C) screening of major driver genes that dominate malignant behavior in cluster 12; (D) the overall expression intensity of target genes; (E) single cell re‐clustering of cancer fibroblast subsets; (F, G): The cell type re‐annotation of cancer fibroblast subsets.

We then focused on cluster 12, which consisted mainly of fibroblasts with EMT characteristics that were more enriched in comparison with the other subgroups (Figure 4A). Single‐cell trajectory and regrouping analyses of cluster 12 were performed to identify potential differences in molecules driving the malignant EMT phenotype in the tumor microenvironment between lymphatic and non‐lymphatic metastatic penile cancer. Pseudo‐time trajectory analysis of cell differentiation fate identified five cell states (i.e., state 1, state 2, state 3, state 4, and state 5), which consisted of one root, two nodes, and four branches (Figure 4B). Multi‐tool enrichment analysis was performed on the upregulated gene set in cluster 12 to delineate its functional role (Figure S4). Noteworthy was that the preferred cell fate decision was state 3 or 4 in lymphatic metastatic penile cancer and state 5 in non‐lymphatic metastatic penile cancer, suggesting that states 3 and 4 were the directions of malignancy. We identified the top 100 genes capable of determining the cell differentiation fate in cluster 12, 87 of which were significant between states 1, 2, 3, 4, and states 1, 5, trajectory branch‐dependent genes for states 3 and 4, and significantly upregulated genes, which intersected on a Venn diagram. Next, differential analysis of cells in cluster 12 was performed to confirm expression of 12 marker genes (*COL1A1*, *COL3A1*, *COL1A2*, *SPARC*, *ACTB*, *IFITM1*, *IFITM3*, *MT‐RNR1*, *MT‐RNR2*, *MT‐ND1*, *MT‐CO2*, and *MT‐CO3*) obtained by analysis of interactive Venn diagrams (Figure 4C). The enriched EMT malignant status seen in cluster 12 suggested that the above‐mentioned genes were important for lymphatic metastasis and acted on fibroblasts in such a way to shape the EMT‐related microenvironment. Based on the previous literature,^34^, ^35^ cluster 12 was divided further into myofibroblastic cancer‐associated fibroblast (CAF) (*TAGLN*, *MYL9*, *MMP11*, and *POSTN*), inflammatory CAF (*CXCL2*, *PDGFRA*, *CXCL12*, and *DPT*), antigen‐presenting CAF (*HLA‐DRA* and *HLA‐DRB1*) and unidentified CAF (Figure 4E–G), and the overall expression of the 12 genes was analyzed (Figure 4D). Upregulated expression of these 12 genes was mainly in myofibroblastic CAF, which suggested that myofibroblastic CAF plays a dominant role in malignant progression.

### 3.5 Cluster 14 demonstrated varying degrees of angiogenesis in the single‐cell tumor landscape

**FIGURE 5:**
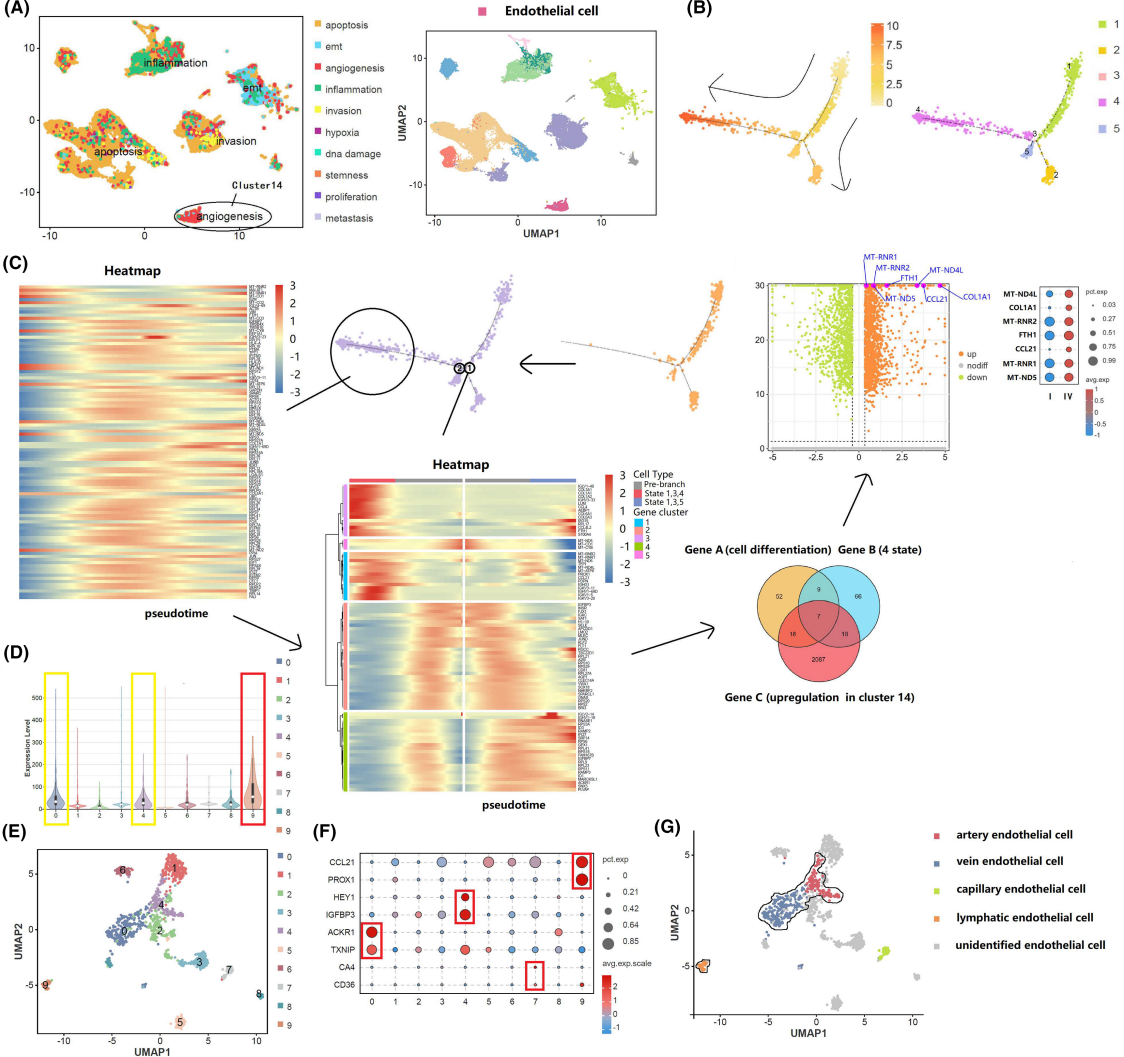
Cluster 14 demonstrated varying degrees of angiogenesis in the single‐cell tumor landscape. (A) Cell malignant status analysis of cluster 14; (B) constructing single cell trajectories in cluster 14; (C) screening of major driver genes that dominate malignant behavior in cluster 14; (D) the overall expression intensity of target genes: (E) single cell re‐clustering of cancer endothelial cell: (F, G) the cell type re‐annotation of cancer endothelial cell subsets.

Next, we focused on cluster 14, which consisted mainly of ECs that had more enriched characteristics of angiogenesis when compared with the other subgroups (Figure 5A). Single‐cell trajectory and regrouping analyses were performed to identify the potential molecules in cluster 14 driving the malignancy‐associated angiogenesis phenotype and metastasis‐associated cells in the tumor microenvironment of lymphatic metastatic and non‐lymphatic metastatic penile cancer. A pseudo‐time trajectory analysis of cell differentiation fate in cluster 14 yielded five cell states (1, 2, 3, 4, and 5), which consisted of one root, two nodes, and four branches (Figure 5B). Comprehensive enrichment analyses of the upregulated gene set in cluster 14 were performed to clarify its role in various biological processes and tumor metabolic pathways (Figure S5). Notably, the most common cell fate decision was state 4 for lymphatic metastatic penile cancer and state 2 for non‐lymphatic metastatic penile cancer, indicating that state 4 was the cell state associated with development of malignancy. We identified the top 100 genes with the ability to determine the cell differentiation fate, of which 86 were significant between states 1, 2 and states 1, 3, 4, and 5, trajectory branch‐dependent genes for state 4 and significantly upregulated genes in cluster 14, which intersected on the Venn diagram. Next, differential analysis of cell cluster 14 was used to confirm expression of seven marker genes (*COL1A1*, *FTH1*, *CCL21*, *MT‐ND4L*, *MT‐ND5*, *MT‐RNR1*, and *MT‐RNR2*) obtained from analysis of an interactive Venn diagram (Figure 5C). Given that cluster 14 showed more enriched characteristics of angiogenesis associated with malignancy, we assumed that the marker genes promoted malignant progression by encouraging ECs to shape the angiogenesis‐related microenvironment. Based on previous studies,^32^, ^36^ cluster 14 were further divided into artery EC (*HEY1* and *IGFBP3*), vein EC (*ACKR1* and *TXNIP*), capillary EC (*CA4* and *CD36*), lymphatic EC (*CCL21* and *PROX1*) and unidentified EC (Figure 5E–G), and the overall expression of 7 genes was analyzed (Figure 5D). Upregulated expression of these 7 genes was mainly in lymphatic EC, which provided a clue for tumor lymphatic metastasis.

### 3.6 Communication signals of epithelial cells, fibroblasts, and ECs in the early tumor microenvironment were stronger than those in advanced cancers

**FIGURE 6:**
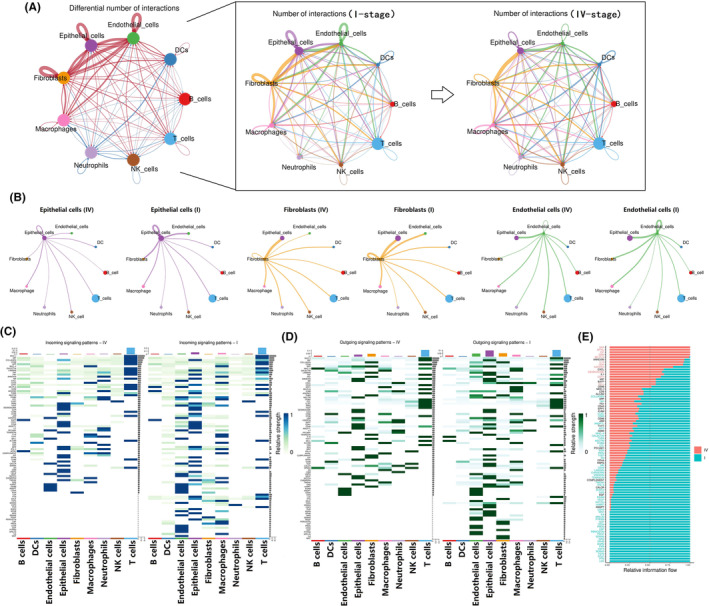
Altered communication signals of tumor microenvironment between early and advanced penile cancers. (A, B) Cell–cell communication in tumor microenvironment of penile cancer; (C) heatmap of incoming signaling in communication signals; (D) heatmap of outcoming signaling in communication signals; (E) evolving signaling pathways in the malignant process of penile cancer.

Analysis of cell–cell communication demonstrated that intercellular signals in epithelial cells, fibroblasts, and ECs were more intense in early penile cancer (Figure 6A,B). The heatmap of incoming signaling and a heatmap of outgoing signaling further confirmed enhanced signaling in epithelial cells, fibroblasts, and ECs in early penile cancer (Figure 6C,D). Enriched signaling in the various pathways identified those that were associated with malignant progression of penile cancer (Figure 6E). Overall, these findings indicate active communication between epithelial cells, fibroblasts, and ECs in the tumor microenvironment, especially in the early stage of penile cancer.

**FIGURE 7:**
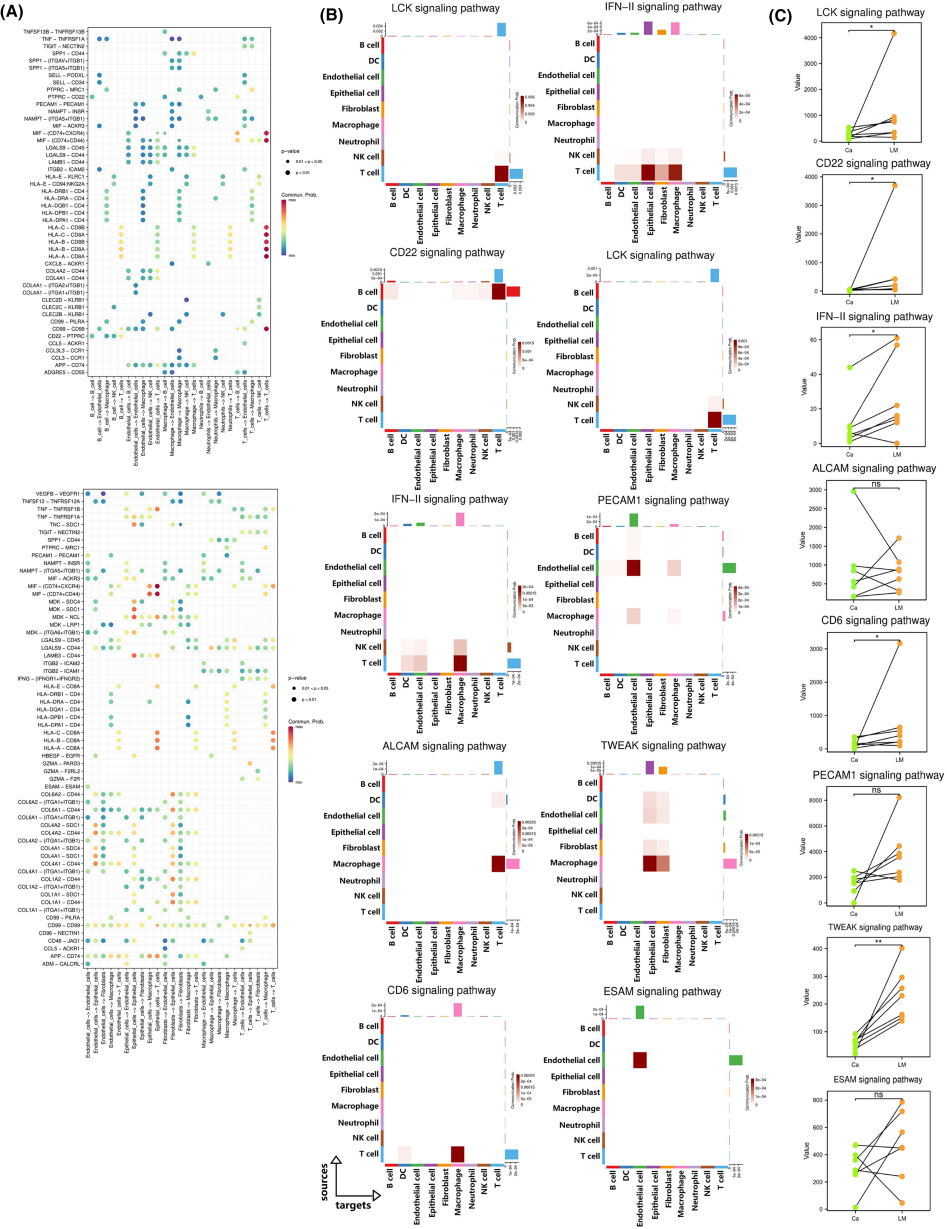
Validation of the existence of premetastatic niche in metastatic lymph nodes of penile cancer. (A) Signaling pathway between the ligand‐receptor cells of the two stage I penile cancers; (B) The top five signaling pathways with the most significant *p*‐values in each sample; (C) Bulk RNA sequencing data from penile cancer and metastatic lymph nodes based on seven advanced patients. (*p < 0.05, **p < 0.01 and ***p < 0.001).

**FIGURE 8:**
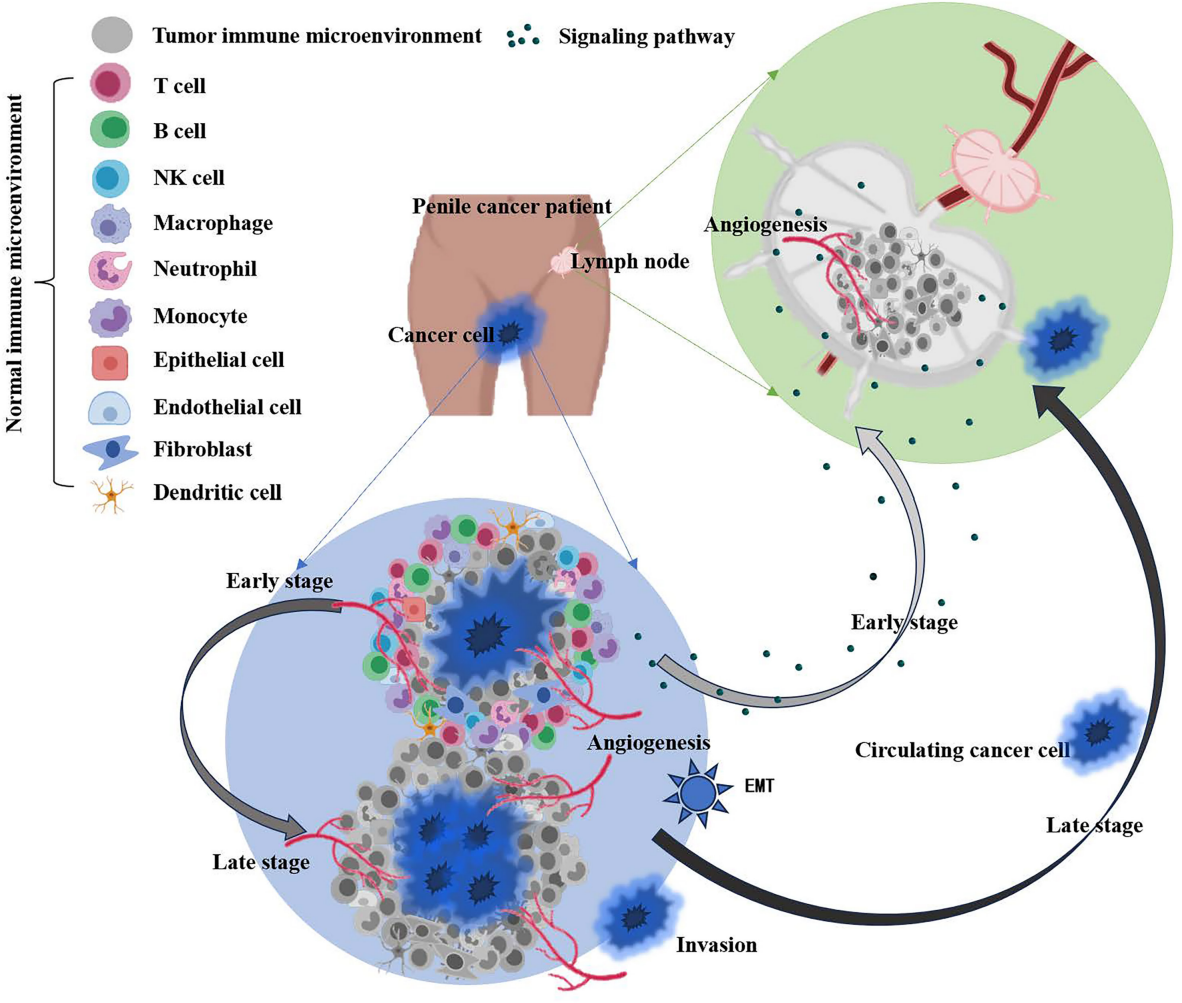
Hypothesis of the existence of premetastatic niche in penile cancer.

Why were there the enhanced cell communication signals in the early penile cancer, which was mainly characterized by the interaction between epithelial cells, fibroblasts and ECs? We speculated that these signals were enhanced in early penile cancer in preparation for lymphatic metastasis. To test this theory, we investigated the signaling pathway between the ligand‐receptor cells in two stage I penile cancer tissues (Figure 7A), and identified the top five signaling pathways with the most significant *p*‐values in both two stage I cancer samples. (Figure 7B). Seven pairs of bulk RNA sequencing data from penile cancer and metastatic lymph nodes from patients with advanced disease showed that signals that were highly expressed in early‐stage cancers were also highly expressed in metastatic lymph nodes, suggesting the metastatic lymph nodes and early tumor cells had similar intercellular signaling pathways (Figure 7C). Therefore, it is reasonable to believe that before the escape of cancer cells to lymph nodes, pre‐metastatic lymph nodes already have a tumor microenvironment similar to that of the primary cancer. Thus, in the early stage, the tumor microenvironment of the primary tumor may help to create a similar microenvironment in a lymph node via certain intercellular signals, creating suitable conditions for migration of cancer cells (Figure 8). This microenvironment is known as the premetastatic niche.

## 4 DISCUSSION

In view of the rarity and poor prognosis of penile cancer and the lack of curative therapy when the disease is advanced, it is essential to understand the potential biological mechanisms of penile cancer and to be able to identify patients at high risk of metastasis as early as possible. Surgery is still the standard treatment for lymph node metastasis of penile cancer, and delayed lymphadenectomy may miss an opportunity for effective treatment.^37^ Therefore, early identification of lymph node metastasis is a focus for researchers in the field of penile cancer worldwide. This study is the first to identify distinctive tumor microenvironments heterogeneity in lymphatic (stage IV) and non‐lymphatic (stage I) metastatic penile cancer at the single‐cell landscape level and to explore the molecular evidence from initiation of cancer to metastatic outgrowth. The tumor microenvironment is a large and complex ecosystem. Its composition and functional status may vary significantly according to cancer type and stage, and its potential role will change with tumor progression and metastasis.^38^ The findings of this study demonstrate that the interaction between epithelial cells, fibroblasts, and ECs and the interplay between invasion, EMT, and angiogenesis are important landscapes in the penile cancer ecosystem.

Once cancer has successfully established a connection between invasion, EMT, and angiogenesis, it can progress to metastasis.^39^, ^40^, ^41^ Invasion is one of the hallmarks of cancer and lays the foundation for dissemination of metastasis. Loss of the adhesion protein E‐cadherin between epithelial cells is central to the invasion process^42^ and is usually accompanied by an EMT‐like transition.^43^ The SERPINB family of genes and *KRT19* can induce invasion by modulating the EMT program.^44^, ^45^ *ELF3* may facilitate invasion of cancer via the phenotypic plasticity of epithelial cells and loss of expression of E‐cadherin,^46^ which would be consistent with our findings in the epithelial cells of cluster 1. Furthermore, for cancer cells to invade surrounding tissues, they need to breach the physical barrier of the basement membrane between the epithelial and stromal cells.^47^ CAFs are key players in remodeling of the basement membrane, helping cancer cells to pass through the basement membrane by secreting protease or applying contractile force.^48^ *S100A* family genes and *LCN2* have been shown to reprogram the epithelial phenotype and promote migration and invasion of cancer cells.^49^, ^50^ Notably, preparation for metastatic spread is also accompanied by changes in the composition and function of CAFs. Abnormal activation or phenotypic transformation of CAFs can lead to development of certain cancers, including breast cancer and gastric cancer.^51^, ^52^ Recent studies have found that CAFs are composed of multiple subtypes that change in a dynamic manner during tumor progression and are regulated at the spatial level.^53^ In this study, we identified four different types of CAFs in penile cancer: myofibroblastic CAF, inflammatory CAF and antigen‐presenting CAF. Myofibroblastic CAFs are important in driving progression of cancer and are significantly associated with poor survival in patients with lung cancer.^54^ Metastasis of cancer through a myofibroblast CAF‐hijacked escape mechanism has been reported.^55^ High expression of *IFITM* family genes has been reported to herald a poor prognosis in patients with renal cancer and enhanced immune infiltration, especially CAFs,^56^ which is similar to our findings for penile cancer in cluster 12. CAFs can also help tumors to evade immune regulation and form an immunosuppressive microenvironment^52^; in short, they are important accomplices of metastasis. Angiogenesis is essential for tumor development. When a tumor has grown in size to 1–2 mm, it needs to establish a vascular system to absorb oxygen and nutrients.^57^ Lack of angiogenesis can limit the progression of some microlesions to invasive carcinoma and cause them to remain dormant.^58^ Tumor‐induced angiogenesis contributes to immunosuppression and immune evasion, and cancer cells can migrate along the luminal surface of blood vessels, which is an important component of metastasis.^59^ A recent study found that biglycan, an angiocrine factor, from tumor ECs stimulates tumor cells to metastasize.^60^ Our study identified four subtypes of EC in penile cancer, namely, artery EC, vein EC, capillary EC, and lymphatic EC. Lymphatic ECs promote lymph node metastasis of lung adenocarcinoma via the *CCL21* signaling axis.^61^ Lymphatic ECs also have important immunomodulatory functions that can attenuate antitumor responses by suppressing T‐cells, including antigen presentation by costimulatory molecules and expression of immune checkpoint molecules.^62^ Interestingly, CAFs are also key coordinators of ECs in tumor angiogenesis.^63^

Importantly, the role of cancer in the host is not limited to phenotypic transformation of the local tumor microenvironment, which, through paracrine effects, creates a microenvironment favorable to cancer in lymph nodes or distant organs before metastasis and spread of cancer cells.^64^ This phenomenon is defined as the premetastatic niche.^65^ Our study found no direct evidence of this phenomenon in penile cancer. However, we indirectly observed two important events that support our hypothesis. First, the degree of cell interaction was more active in early penile cancer, and almost all of the enhanced cell communication signals came from the interaction between epithelial cells, fibroblasts, and ECs. Second, the metastatic lymph nodes and early tumor cells had similar intercellular signaling pathways in the tumor microenvironment. Remodeling of the extracellular matrix, phenotypic alteration of resident epithelial cells, fibroblasts, and ECs, and an immunosuppressive microenvironment are central to generation of the premetastatic niche^38^, ^66^, ^67^, ^68^ and similar to the landscape observed in penile cancer in our study. Furthermore, the initial signals that have been reported to promote formation of the premetastatic niche include cancer‐derived mediators, most notably the *S100* family genes and *LCN2*,^69^, ^70^, ^71^ which is also consistent with our findings.

## 5 CONCLUSIONS

In summary, our study identified the tumor microenvironment heterogeneity that evolved from non‐lymphatic to lymphatic metastatic penile cancer, which provides insights into the mechanisms underlying progression of penile cancer, the premetastatic niche, and lymphatic metastasis.

## AUTHOR CONTRIBUTIONS

**Da‐Ming Xu:** Formal analysis (lead); investigation (lead); writing – original draft (lead); writing – review and editing (lead). **Xiao‐Yu Zhuang:** Formal analysis (lead); investigation (lead); writing – original draft (lead). **Hua‐Li Ma:** Formal analysis (lead); methodology (lead). **Zai‐Shang Li:** Data curation (lead). **Li‐Chao Wei:** Supervision (lead). **Jun‐Hang Luo:** Project administration (lead); writing – review and editing (equal). **Hui Han:** Project administration (lead); writing – review and editing (lead).

## FUNDING INFORMATION

This work was supported by grants from Natural Science Foundation of Guangdong Province (2022A1515012321), Science and Technology Planning Project of Shenzhen Municipality (JCYJ20190807145409328) and Shenzhen Science and Technology Program (RCYX20221008093032008).

## CONFLICT OF INTEREST STATEMENT

The authors declare that they have no competing interests.

## ETHICS APPROVAL AND CONSENT TO PARTICIPATE

This research was approved by the Institutional Review Board of Sun Yat‐sen University Cancer Center (B2023‐390‐01) and conducted in accordance with the criteria set by the Declaration of Helsinki. The study design and conduct complied with all relevant regulations regarding the use of human study participants. Written informed consent was received from all participants.

## Supporting information


Figure S1.

## Data Availability

The data that support the findings of this study are available from the corresponding author upon reasonable request.

## References

[1] Chaffer CL , Weinberg RA . A perspective on cancer cell metastasis. Science. 2011;331(6024):1559‐1564. 21436443 10.1126/science.1203543

[2] Fu L , Tian T , Yao K , et al. Global pattern and trends in penile cancer incidence: population‐based study. JMIR Public Health Surveill. 2022;8(7):e34874. 35793140 10.2196/34874 PMC9301560

[3] Brouwer OR , Albersen M , Parnham A , et al. European Association of Urology‐American Society of Clinical Oncology collaborative guideline on penile cancer: 2023 update. Eur Urol. 2023;83(6):548‐560. 36906413 10.1016/j.eururo.2023.02.027

[4] Thomas A , Necchi A , Muneer A , et al. Penile cancer. Nat Rev Dis Primers. 2021;7(1):11. 33574340 10.1038/s41572-021-00246-5

[5] Sanchez DF , Soares F , Alvarado‐Cabrero I , et al. Pathological factors, behavior, and histological prognostic risk groups in subtypes of penile squamous cell carcinomas (SCC). Semin Diagn Pathol. 2015;32(3):222‐231. 25677263 10.1053/j.semdp.2014.12.017

[6] Horenblas S , van Tinteren H . Squamous cell carcinoma of the penis. IV. Prognostic factors of survival: analysis of tumor, nodes and metastasis classification system. J Urol. 1994;151(5):1239‐1243. 8158767 10.1016/s0022-5347(17)35221-7

[7] Leone A , Diorio GJ , Pettaway C , Master V , Spiess PE . Contemporary management of patients with penile cancer and lymph node metastasis. Nat Rev Urol. 2017;14(6):335‐347. 28401957 10.1038/nrurol.2017.47

[8] Gerstberger S , Jiang Q , Ganesh K . Metastasis. Cell. 2023;186(8):1564‐1579. doi:10.1016/j.cell.2023.03.003 37059065 PMC10511214

[9] Mehlen P , Puisieux A . Metastasis: a question of life or death. Nat Rev Cancer. 2006;6(6):449‐458. doi:10.1038/nrc1886 16723991

[10] Kroon BK , Horenblas S , Lont AP , Tanis PJ , Gallee MP , Nieweg OE . Patients with penile carcinoma benefit from immediate resection of clinically occult lymph node metastases. J Urol. 2005;173(3):816‐819. 15711276 10.1097/01.ju.0000154565.37397.4d

[11] Leijte JA , Graafland NM , Valdés Olmos RA , van Boven HH , Hoefnagel CA , Horenblas S . Prospective evaluation of hybrid 18F‐fluorodeoxyglucose positron emission tomography/computed tomography in staging clinically node‐negative patients with penile carcinoma. BJU Int. 2009;104(5):640‐644. 19281465 10.1111/j.1464-410X.2009.08450.x

[12] Joshi VB , Spiess PE , Necchi A , Pettaway CA , Chahoud J . Immune‐based therapies in penile cancer. Nat Rev Urol. 2022;19(8):457‐474. 35851333 10.1038/s41585-022-00617-x

[13] Maisch P , Koll F , Bolenz C , Chun FK , Gschwend JE , Schmid SC . Combination of radiation and immunotherapy in the treatment of genitourinary malignancies: a systematic review and meta‐analysis. Urol Oncol. 2023;41(5):219‐232. 36372634 10.1016/j.urolonc.2022.10.009

[14] Wei SC , Duffy CR , Allison JP . Fundamental mechanisms of immune checkpoint blockade therapy. Cancer Discov. 2018;8(9):1069‐1086. 30115704 10.1158/2159-8290.CD-18-0367

[15] Mempel TR , Henrickson SE , Von Andrian UH . T‐cell priming by dendritic cells in lymph nodes occurs in three distinct phases. Nature. 2004;427(6970):154‐159. 14712275 10.1038/nature02238

[16] Hanahan D , Weinberg RA . Hallmarks of cancer: the next generation. Cell. 2011;144(5):646‐674. 21376230 10.1016/j.cell.2011.02.013

[17] Lun ATL , Riesenfeld S , Andrews T , et al. EmptyDrops: distinguishing cells from empty droplets in droplet‐based single‐cell RNA sequencing data. Genome Biol. 2019;20(1):63. 30902100 10.1186/s13059-019-1662-y PMC6431044

[18] Butler A , Hoffman P , Smibert P , Papalexi E , Satija R . Integrating single‐cell transcriptomic data across different conditions, technologies, and species. Nat Biotechnol. 2018;36(5):411‐420. 29608179 10.1038/nbt.4096 PMC6700744

[19] McGinnis CS , Murrow LM , Gartner ZJ . DoubletFinder: doublet detection in single‐cell RNA sequencing data using artificial nearest neighbors. Cell Syst. 2019;8(4):329‐337.e4. 30954475 10.1016/j.cels.2019.03.003 PMC6853612

[20] Korsunsky I , Millard N , Fan J , et al. Fast, sensitive and accurate integration of single‐cell data with harmony. Nat Methods. 2019;16(12):1289‐1296. 31740819 10.1038/s41592-019-0619-0 PMC6884693

[21] Aran D , Looney AP , Liu L , et al. Reference‐based analysis of lung single‐cell sequencing reveals a transitional profibrotic macrophage. Nat Immunol. 2019;20(2):163‐172. 30643263 10.1038/s41590-018-0276-y PMC6340744

[22] Ashburner M , Ball CA , Blake JA , et al. Gene ontology: tool for the unification of biology. The Gene Ontology Consortium. Nat Genet. 2000;25(1):25‐29. 10802651 10.1038/75556 PMC3037419

[23] Kanehisa M , Goto S . KEGG: kyoto encyclopedia of genes and genomes. Nucleic Acids Res. 2000;28(1):27‐30. 10592173 10.1093/nar/28.1.27 PMC102409

[24] Schriml LM , Mitraka E , Munro J , et al. Human disease ontology 2018 update: classification, content and workflow expansion. Nucleic Acids Res. 2019;47(D1):D955‐D962. 30407550 10.1093/nar/gky1032 PMC6323977

[25] Fabregat A , Jupe S , Matthews L , et al. The Reactome pathway knowledgebase. Nucleic Acids Res. 2018;46(D1):D649‐D655. 29145629 10.1093/nar/gkx1132 PMC5753187

[26] Macosko EZ , Basu A , Satija R , et al. Highly parallel genome‐wide expression profiling of individual cells using Nanoliter droplets. Cell. 2015;161(5):1202‐1214. 26000488 10.1016/j.cell.2015.05.002 PMC4481139

[27] Neftel C , Laffy J , Filbin MG , et al. An integrative model of cellular states, plasticity, and genetics for glioblastoma. Cell. 2019;178(4):835‐849.e21. 31327527 10.1016/j.cell.2019.06.024 PMC6703186

[28] Yuan H , Yan M , Zhang G , et al. CancerSEA: a cancer single‐cell state atlas. Nucleic Acids Res. 2019;47(D1):D900‐D908. 30329142 10.1093/nar/gky939 PMC6324047

[29] Trapnell C , Cacchiarelli D , Grimsby J , et al. The dynamics and regulators of cell fate decisions are revealed by pseudotemporal ordering of single cells. Nat Biotechnol. 2014;32(4):381‐386. 24658644 10.1038/nbt.2859 PMC4122333

[30] Qiu X , Hill A , Packer J , Lin D , Ma YA , Trapnell C . Single‐cell mRNA quantification and differential analysis with census. Nat Methods. 2017;14(3):309‐315. 28114287 10.1038/nmeth.4150 PMC5330805

[31] Jin S , Guerrero‐Juarez CF , Zhang L , et al. Inference and analysis of cell‐cell communication using CellChat. Nat Commun. 2021;12(1):1088. 33597522 10.1038/s41467-021-21246-9 PMC7889871

[32] Zhao L , Han S , Su H , et al. Single‐cell transcriptome atlas of the human corpus cavernosum. Nat Commun. 2022;13(1):4302. 35879305 10.1038/s41467-022-31950-9 PMC9314400

[33] Chumduri C , Gurumurthy RK , Berger H , et al. Opposing Wnt signals regulate cervical squamocolumnar homeostasis and emergence of metaplasia. Nat Cell Biol. 2021;23(2):184‐197. doi:10.1038/s41556-020-00619-0 33462395 PMC7878191

[34] Elyada E , Bolisetty M , Laise P , et al. Cross‐species single‐cell analysis of pancreatic ductal adenocarcinoma reveals antigen‐presenting cancer‐associated fibroblasts. Cancer Discov. 2019;9(8):1102‐1123. 31197017 10.1158/2159-8290.CD-19-0094 PMC6727976

[35] Solé‐Boldo L , Raddatz G , Schütz S , et al. Single‐cell transcriptomes of the human skin reveal age‐related loss of fibroblast priming. Commun Biol. 2020;3(1):188. 32327715 10.1038/s42003-020-0922-4 PMC7181753

[36] Geldhof V , de Rooij LPMH , Sokol L , et al. Single cell atlas identifies lipid‐processing and immunomodulatory endothelial cells in healthy and malignant breast. Nat Commun. 2022;13(1):5511. 36127427 10.1038/s41467-022-33052-y PMC9489707

[37] Sachdeva A , McGuinness L , Zapala Ł , et al. Management of lymph node‐positive penile cancer: a systematic review. Eur Urol. 2024;85(3):257‐273. doi:10.1016/j.eururo.2023.04.018 37208237

[38] de Visser KE , Joyce JA . The evolving tumor microenvironment: from cancer initiation to metastatic outgrowth. Cancer Cell. 2023;41(3):374‐403. doi:10.1016/j.ccell.2023.02.016 36917948

[39] Yilmaz M , Christofori G . EMT, the cytoskeleton, and cancer cell invasion. Cancer Metastasis Rev. 2009;28(1–2):15‐33. 19169796 10.1007/s10555-008-9169-0

[40] Unterleuthner D , Neuhold P , Schwarz K , et al. Cancer‐associated fibroblast‐derived WNT2 increases tumor angiogenesis in colon cancer. Angiogenesis. 2020;23(2):159‐177. 31667643 10.1007/s10456-019-09688-8 PMC7160098

[41] Ng L , Wong SK , Huang Z , et al. CD26 induces colorectal cancer angiogenesis and metastasis through CAV1/MMP1 signaling. Int J Mol Sci. 2022;23(3):1181. 35163100 10.3390/ijms23031181 PMC8835326

[42] Mendonsa AM , Na TY , Gumbiner BM . E‐cadherin in contact inhibition and cancer. Oncogene. 2018;37(35):4769‐4780. 29780167 10.1038/s41388-018-0304-2 PMC6119098

[43] Nieto MA , Huang RY , Jackson RA , Thiery JP . EMT: 2016. Cell. 2016;166(1):21‐45. 27368099 10.1016/j.cell.2016.06.028

[44] Quarta S , Vidalino L , Turato C , et al. SERPINB3 induces epithelial‐mesenchymal transition. J Pathol. 2010;221(3):343‐356. 20527027 10.1002/path.2708

[45] Wang W , He J , Lu H , Kong Q , Lin S . KRT8 and KRT19, associated with EMT, are hypomethylated and overexpressed in lung adenocarcinoma and link to unfavorable prognosis. Biosci Rep. 2020;40(7):BSR20193468. 32519739 10.1042/BSR20193468 PMC7335829

[46] Zheng L , Xu M , Xu J , et al. ELF3 promotes epithelial‐mesenchymal transition by protecting ZEB1 from miR‐141‐3p‐mediated silencing in hepatocellular carcinoma. Cell Death Dis. 2018;9(3):387. 29523781 10.1038/s41419-018-0399-y PMC5845010

[47] Chang TT , Thakar D , Weaver VM . Force‐dependent breaching of the basement membrane. Matrix Biol. 2017;57‐58:178‐189. 10.1016/j.matbio.2016.12.005 PMC5328923 28025167

[48] Chang J , Chaudhuri O . Beyond proteases: basement membrane mechanics and cancer invasion. J Cell Biol. 2019;218(8):2456‐2469. 31315943 10.1083/jcb.201903066 PMC6683740

[49] Santiago‐Sánchez GS , Pita‐Grisanti V , Quiñones‐Díaz B , Gumpper K , Cruz‐Monserrate Z , Vivas‐Mejía PE . Biological functions and therapeutic potential of Lipocalin 2 in cancer. Int J Mol Sci. 2020;21(12):4365. 32575507 10.3390/ijms21124365 PMC7352275

[50] Jo SH , Heo WH , Son HY , et al. S100A8/A9 mediate the reprograming of normal mammary epithelial cells induced by dynamic cell‐cell interactions with adjacent breast cancer cells. Sci Rep. 2021;11(1):1337. 33446797 10.1038/s41598-020-80625-2 PMC7809201

[51] Li X , Sun Z , Peng G , et al. Single‐cell RNA sequencing reveals a pro‐invasive cancer‐associated fibroblast subgroup associated with poor clinical outcomes in patients with gastric cancer. Theranostics. 2022;12(2):620‐638. 34976204 10.7150/thno.60540 PMC8692898

[52] Costa A , Kieffer Y , Scholer‐Dahirel A , et al. Fibroblast heterogeneity and immunosuppressive environment in human breast cancer. Cancer Cell. 2018;33(3):463‐479. e10. 29455927 10.1016/j.ccell.2018.01.011

[53] Sahai E , Astsaturov I , Cukierman E , et al. A framework for advancing our understanding of cancer‐associated fibroblasts. Nat Rev Cancer. 2020;20(3):174‐186. 31980749 10.1038/s41568-019-0238-1 PMC7046529

[54] Tang PC , Chung JY , Xue VW , et al. Smad3 promotes cancer‐associated fibroblasts generation via macrophage‐Myofibroblast transition. Adv Sci (Weinh). 2022;9(1):e2101235. 34791825 10.1002/advs.202101235 PMC8728853

[55] Sun X , He X , Zhang Y , et al. Inflammatory cell‐derived CXCL3 promotes pancreatic cancer metastasis through a novel myofibroblast‐hijacked cancer escape mechanism. Gut. 2022;71(1):129‐147. 33568427 10.1136/gutjnl-2020-322744

[56] Xu Y , Huang D , Zhang K , et al. Overexpressing IFITM family genes predict poor prognosis in kidney renal clear cell carcinoma. Transl Androl Urol. 2021;10(10):3837‐3851. 34804826 10.21037/tau-21-848 PMC8575577

[57] Folkman J . Tumor angiogenesis: therapeutic implications. N Engl J Med. 1971;285(21):1182‐1186. 4938153 10.1056/NEJM197111182852108

[58] Naumov GN , Folkman J , Straume O . Tumor dormancy due to failure of angiogenesis: role of the microenvironment. Clin Exp Metastasis. 2009;26(1):51‐60. 18563595 10.1007/s10585-008-9176-0

[59] Latacz E , Caspani E , Barnhill R , et al. Pathological features of vessel co‐option versus sprouting angiogenesis. Angiogenesis. 2020;23(1):43‐54. 31655928 10.1007/s10456-019-09690-0

[60] Maishi N , Hida K . Tumor endothelial cells accelerate tumor metastasis. Cancer Sci. 2017;108(10):1921‐1926. 28763139 10.1111/cas.13336 PMC5623747

[61] Zhang S , Wang H , Xu Z , Bai Y , Xu L . Lymphatic metastasis of NSCLC involves chemotaxis effects of lymphatic endothelial cells through the CCR7‐CCL21 Axis modulated by TNF‐α. Genes (Basel). 2020;11(11):1309. 33158173 10.3390/genes11111309 PMC7694274

[62] Petrova TV , Koh GY . Biological functions of lymphatic vessels. Science. 2020;369(6500):eaax4063. 32646971 10.1126/science.aax4063

[63] Egeblad M , Rasch MG , Weaver VM . Dynamic interplay between the collagen scaffold and tumor evolution. Curr Opin Cell Biol. 2010;22(5):697‐706. 20822891 10.1016/j.ceb.2010.08.015 PMC2948601

[64] Peinado H , Zhang H , Matei IR , et al. Pre‐metastatic niches: organ‐specific homes for metastases. Nat Rev Cancer. 2017;17(5):302‐317. 28303905 10.1038/nrc.2017.6

[65] Lu Z , Zou J , Li S , et al. Epigenetic therapy inhibits metastases by disrupting premetastatic niches. Nature. 2020;579(7798):284‐290. 32103175 10.1038/s41586-020-2054-x PMC8765085

[66] Wang Z , Zhu J , Liu Y , Wang Z , Cao X , Gu Y . Tumor‐polarized GPX3+ AT2 lung epithelial cells promote premetastatic niche formation. Proc Natl Acad Sci USA. 2022;119(32):e2201899119. 35914155 10.1073/pnas.2201899119 PMC9371733

[67] Zeng H , Hou Y , Zhou X , et al. Cancer‐associated fibroblasts facilitate premetastatic niche formation through lncRNA SNHG5‐mediated angiogenesis and vascular permeability in breast cancer. Theranostics. 2022;12(17):7351‐7370. 36438499 10.7150/thno.74753 PMC9691361

[68] Zhang G , Li M , Zhou D , Yang X , Zhang W , Gao R . Loss of endothelial EMCN drives tumor lung metastasis through the premetastatic niche. J Transl Med. 2022;20(1):446. 36184589 10.1186/s12967-022-03649-4 PMC9528146

[69] Sakaguchi M . S100‐SPECT uncovers cellular and molecular events of pre‐metastatic niche formation and following organ‐specific cancer metastasis. Theranostics. 2017;7(10):2649‐2651. 28819453 10.7150/thno.19866 PMC5558559

[70] Chung YH , Ortega‐Rivera OA , Volckaert BA , Jung E , Zhao Z , Steinmetz NF . Viral nanoparticle vaccines against S100A9 reduce lung tumor seeding and metastasis. Proc Natl Acad Sci USA. 2023;120(43):e2221859120. 37844250 10.1073/pnas.2221859120 PMC10614828

[71] Meade KJ , Sanchez F , Aguayo A , et al. Secretomes from metastatic breast cancer cells, enriched for a prognostically unfavorable LCN2 axis, induce anti‐inflammatory MSC actions and a tumor‐supportive premetastatic lung. Oncotarget. 2019;10(32):3027‐3039. 31105883 10.18632/oncotarget.26903 PMC6508963

