# Supplementary material for: Altered tumor microenvironment heterogeneity of penile cancer during progression from non‐lymphatic to lymphatic metastasis
Source: Cancer Med. 2024 Jul 14;13(14):e70025. doi: 10.1002/cam4.70025 (PMC11246611; doi:10.1002/cam4.70025)
Supplement: Supplementary file 1 — Figure S1. [file CAM4-13-e70025-s001.pdf]

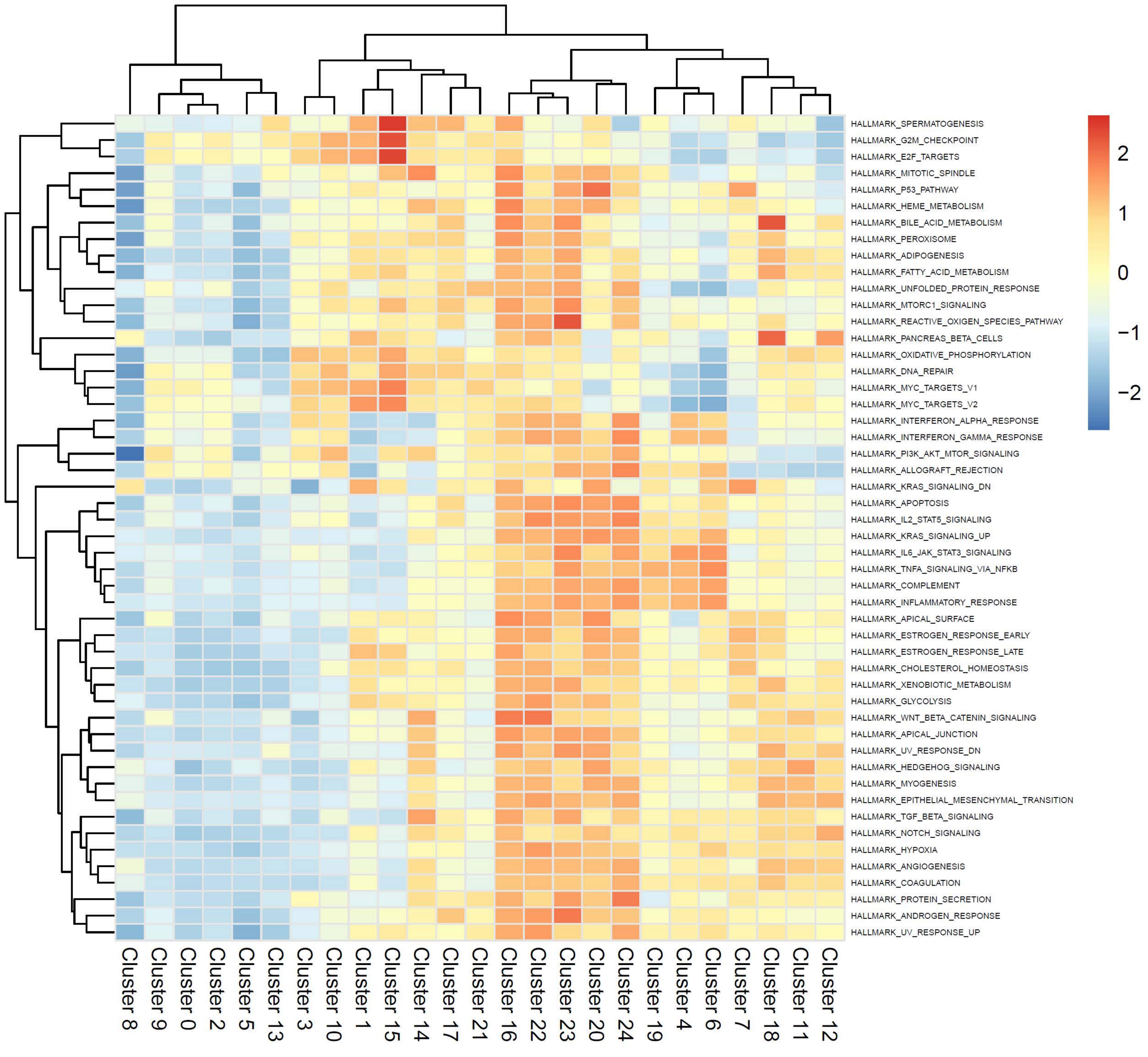

HALLMARK_SPERMATOGENESIS
HALLMARK_G2M_CHECKPOINT
HALLMARK_E2F_TARGETS
HALLMARK_MITOTIC_SPINDLE
HALLMARK_P53_PATHWAY
HALLMARK_HEME_METABOLISM
HALLMARK_BILE_ACID_METABOLISM
HALLMARK_PEROXISOME
HALLMARK_ADIPOGENESIS
HALLMARK_FATTY_ACID_METABOLISM
HALLMARK_UNFOLDED_PROTEIN_RESPONSE
HALLMARK_MTORC1_SIGNALING
HALLMARK_REACTIVE_OXIGEN_SPECIES_PATHWAY
HALLMARK_PANCREAS_BETA_CELLS
HALLMARK_OXIDATIVE_PHOSPHORYLATION
HALLMARK_DNA_REPAIR
HALLMARK_MYC_TARGETS_V1
HALLMARK_MYC_TARGETS_V2
HALLMARK_INTERFERON_ALPHA_RESPONSE
HALLMARK_INTERFERON_GAMMA_RESPONSE
HALLMARK_PI3K_AKT_MTOR_SIGNALING
HALLMARK_ALLOGRAFT_REJECTION
HALLMARK_KRAS_SIGNALING_DN
HALLMARK_APOPTOSIS
HALLMARK_IL2_STAT5_SIGNALING
HALLMARK_KRAS_SIGNALING_UP
HALLMARK_IL6_JAK_STAT3_SIGNALING
HALLMARK_TNFA_SIGNALING_VIA_NFKB
HALLMARK_COMPLEMENT
HALLMARK_INFLAMMATORY_RESPONSE
HALLMARK_APICAL_SURFACE
HALLMARK_ESTROGEN_RESPONSE_EARLY
HALLMARK_ESTROGEN_RESPONSE_LATE
HALLMARK_CHOLESTEROL_HOMEOSTASIS
HALLMARK_XENOBIOTIC_METABOLISM
HALLMARK_GLYCOLYSIS
HALLMARK_WNT_BETA_CATENIN_SIGNALING
HALLMARK_APICAL_JUNCTION
HALLMARK_UV_RESPONSE_DN
HALLMARK_HEDGEHOG_SIGNALING
HALLMARK_MYOGENESIS
HALLMARK_EPITHELIAL_MESENCHYMAL_TRANSITION
HALLMARK_TGF_BETA_SIGNALING
HALLMARK_NOTCH_SIGNALING
HALLMARK_HYPOXIA
HALLMARK_ANGIOGENESIS
HALLMARK_COAGULATION
HALLMARK_PROTEIN_SECRETION
HALLMARK_ANDROGEN_RESPONSE
HALLMARK_UV_RESPONSE_UP
2
1
0
−1
−2
Cluster 8
Cluster 9
Cluster 0
Cluster 2
Cluster 5
Cluster 13
Cluster 3
Cluster 10
Cluster 1
Cluster 15
Cluster 14
Cluster 17
Cluster 21
Cluster 16
Cluster 22
Cluster 23
Cluster 20
Cluster 24
Cluster 19
Cluster 4
Cluster 6
Cluster 7
Cluster 18
Cluster 11
Cluster 12

Supplementary 1. Heatmap of GSVA analysis of distinct clusters (hallmark gene sets)

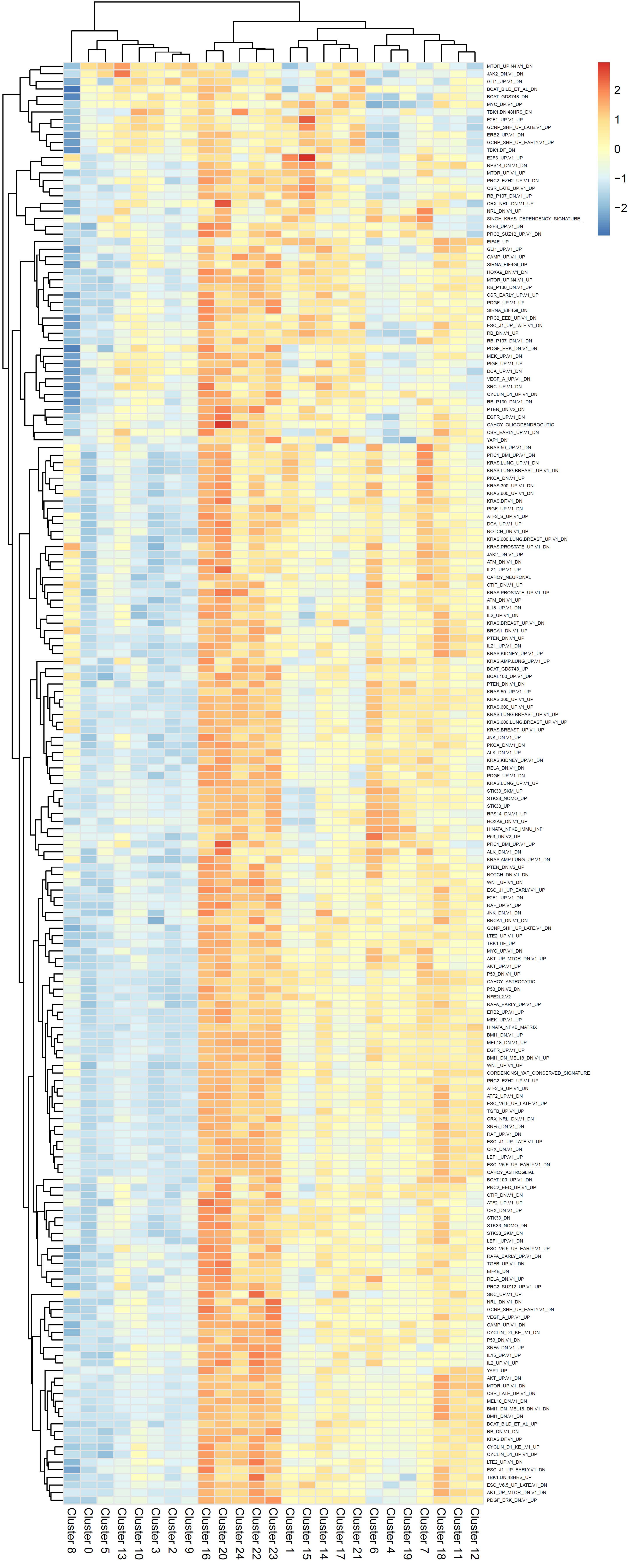
2
1
0
−1
−2
MTOR_UP.N4.V1_DN
JAK2_DN.V1_DN
GLI1_UP.V1_DN
BCAT_BILD_ET_AL_DN
BCAT_GDS748_DN
MYC_UP.V1_UP
TBK1.DN.48HRS_DN
E2F1_UP.V1_UP
GCNP_SHH_UP_LATE.V1_UP
ERB2_UP.V1_DN
GCNP_SHH_UP_EARLY.V1_UP
TBK1.DF_DN
E2F3_UP.V1_UP
RPS14_DN.V1_DN
MTOR_UP.V1_UP
PRC2_EZH2_UP.V1_DN
CSR_LATE_UP.V1_UP
RB_P107_DN.V1_UP
CRX_NRL_DN.V1_UP
NRL_DN.V1_UP
SINGH_KRAS_DEPENDENCY_SIGNATURE_
E2F3_UP.V1_DN
PRC2_SUZ12_UP.V1_DN
EIF4E_UP
GLI1_UP.V1_UP
CAMP_UP.V1_UP
SIRNA_EIF4GI_UP
HOXA9_DN.V1_DN
MTOR_UP.N4.V1_UP
RB_P130_DN.V1_UP
CSR_EARLY_UP.V1_UP
PDGF_UP.V1_UP
SIRNA_EIF4GI_DN
PRC2_EED_UP.V1_DN
ESC_J1_UP_LATE.V1_DN
RB_DN.V1_UP
RB_P107_DN.V1_DN
PDGF_ERK_DN.V1_DN
MEK_UP.V1_DN
PIGF_UP.V1_UP
DCA_UP.V1_DN
VEGF_A_UP.V1_DN
SRC_UP.V1_DN
CYCLIN_D1_UP.V1_DN
RB_P130_DN.V1_DN
PTEN_DN.V2_DN
EGFR_UP.V1_DN
CAHOY_OLIGODENDROCUTIC
CSR_EARLY_UP.V1_DN
YAP1_DN
KRAS.50_UP.V1_DN
PRC1_BMI_UP.V1_DN
KRAS.LUNG_UP.V1_DN
KRAS.LUNG.BREAST_UP.V1_DN
PKCA_DN.V1_UP
KRAS.300_UP.V1_DN
KRAS.600_UP.V1_DN
KRAS.DF.V1_DN
PIGF_UP.V1_DN
ATF2_S_UP.V1_UP
DCA_UP.V1_UP
NOTCH_DN.V1_UP
KRAS.600.LUNG.BREAST_UP.V1_DN
KRAS.PROSTATE_UP.V1_DN
JAK2_DN.V1_UP
ATM_DN.V1_DN
IL21_UP.V1_UP
CAHOY_NEURONAL
CTIP_DN.V1_UP
KRAS.PROSTATE_UP.V1_UP
ATM_DN.V1_UP
IL15_UP.V1_DN
IL2_UP.V1_DN
KRAS.BREAST_UP.V1_DN
BRCA1_DN.V1_UP
PTEN_DN.V1_UP
IL21_UP.V1_DN
KRAS.KIDNEY_UP.V1_UP
KRAS.AMP.LUNG_UP.V1_UP
BCAT_GDS748_UP
BCAT.100_UP.V1_UP
PTEN_DN.V1_DN
KRAS.50_UP.V1_UP
KRAS.300_UP.V1_UP
KRAS.600_UP.V1_UP
KRAS.LUNG.BREAST_UP.V1_UP
KRAS.600.LUNG.BREAST_UP.V1_UP
KRAS.BREAST_UP.V1_UP
JNK_DN.V1_UP
PKCA_DN.V1_DN
ALK_DN.V1_UP
KRAS.KIDNEY_UP.V1_DN
RELA_DN.V1_DN
PDGF_UP.V1_DN
KRAS.LUNG_UP.V1_UP
STK33_SKM_UP
STK33_NOMO_UP
STK33_UP
RPS14_DN.V1_UP
HOXA9_DN.V1_UP
HINATA_NFKB_IMMU_INF
P53_DN.V2_UP
PRC1_BMI_UP.V1_UP
ALK_DN.V1_DN
KRAS.AMP.LUNG_UP.V1_DN
PTEN_DN.V2_UP
NOTCH_DN.V1_DN
WNT_UP.V1_DN
ESC_J1_UP_EARLY.V1_UP
E2F1_UP.V1_DN
RAF_UP.V1_UP
JNK_DN.V1_DN
BRCA1_DN.V1_DN
GCNP_SHH_UP_LATE.V1_DN
LTE2_UP.V1_UP
TBK1.DF_UP
MYC_UP.V1_DN
AKT_UP_MTOR_DN.V1_UP
AKT_UP.V1_UP
P53_DN.V1_UP
CAHOY_ASTROCYTIC
P53_DN.V2_DN
NFE2L2.V2
RAPA_EARLY_UP.V1_UP
ERB2_UP.V1_UP
MEK_UP.V1_UP
HINATA_NFKB_MATRIX
BMI1_DN.V1_UP
MEL18_DN.V1_UP
EGFR_UP.V1_UP
BMI1_DN_MEL18_DN.V1_UP
WNT_UP.V1_UP
CORDENONSI_YAP_CONSERVED_SIGNATURE
PRC2_EZH2_UP.V1_UP
ATF2_S_UP.V1_DN
ATF2_UP.V1_DN
ESC_V6.5_UP_LATE.V1_UP
TGFB_UP.V1_UP
CRX_NRL_DN.V1_DN
SNF5_DN.V1_DN
RAF_UP.V1_DN
ESC_J1_UP_LATE.V1_UP
CRX_DN.V1_DN
LEF1_UP.V1_UP
ESC_V6.5_UP_EARLY.V1_DN
CAHOY_ASTROGLIAL
BCAT.100_UP.V1_DN
PRC2_EED_UP.V1_UP
CTIP_DN.V1_DN
ATF2_UP.V1_UP
CRX_DN.V1_UP
STK33_DN
STK33_NOMO_DN
STK33_SKM_DN
LEF1_UP.V1_DN
ESC_V6.5_UP_EARLY.V1_UP
RAPA_EARLY_UP.V1_DN
TGFB_UP.V1_DN
EIF4E_DN
RELA_DN.V1_UP
PRC2_SUZ12_UP.V1_UP
SRC_UP.V1_UP
NRL_DN.V1_DN
GCNP_SHH_UP_EARLY.V1_DN
VEGF_A_UP.V1_UP
CAMP_UP.V1_DN
CYCLIN_D1_KE_.V1_DN
P53_DN.V1_DN
SNF5_DN.V1_UP
IL15_UP.V1_UP
IL2_UP.V1_UP
YAP1_UP
AKT_UP.V1_DN
MTOR_UP.V1_DN
CSR_LATE_UP.V1_DN
MEL18_DN.V1_DN
BMI1_DN_MEL18_DN.V1_DN
BMI1_DN.V1_DN
BCAT_BILD_ET_AL_UP
RB_DN.V1_DN
KRAS.DF.V1_UP
CYCLIN_D1_KE_.V1_UP
CYCLIN_D1_UP.V1_UP
LTE2_UP.V1_DN
ESC_J1_UP_EARLY.V1_DN
TBK1.DN.48HRS_UP
ESC_V6.5_UP_LATE.V1_DN
AKT_UP_MTOR_DN.V1_DN
PDGF_ERK_DN.V1_UP
Cluster 8
Cluster 0
Cluster 5
Cluster 13
Cluster 10
Cluster 3
Cluster 2
Cluster 9
Cluster 16
Cluster 20
Cluster 24
Cluster 22
Cluster 23
Cluster 1
Cluster 15
Cluster 14
Cluster 17
Cluster 21
Cluster 6
Cluster 4
Cluster 19
Cluster 7
Cluster 18
Cluster 11
Cluster 12

Supplementary 2. Heatmap of GSVA analysis of distinct clusters (oncogenic signature gene sets)

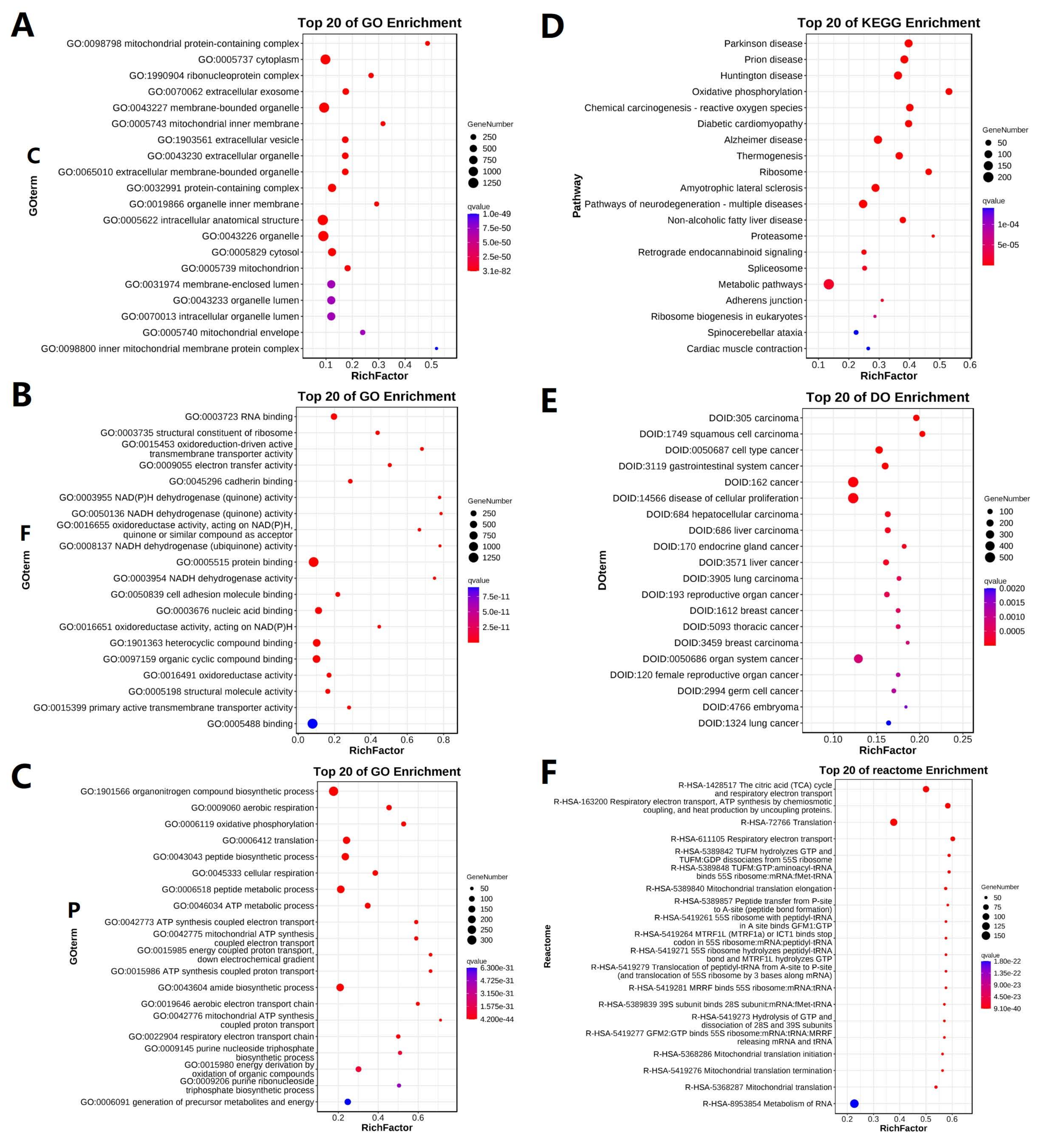
A
Top 20 of GO Enrichment
GO:0098798 mitochondrial protein-containing complex
GO:0005737 cytoplasm
GO:1990904 ribonucleoprotein complex
GO:0070062 extracellular exosome
GO:0043227 membrane-bounded organelle
GO:0005743 mitochondrial inner membrane
GO:1903561 extracellular vesicle
GO:0043230 extracellular organelle
GO:0065010 extracellular membrane-bounded organelle
GO:0032991 protein-containing complex
GO:0019866 organelle inner membrane
GO:0005622 intracellular anatomical structure
GO:0043226 organelle
GO:0005829 cytosol
GO:0005739 mitochondrion
GO:0031974 membrane-enclosed lumen
GO:0043233 organelle lumen
GO:0070013 intracellular organelle lumen
GO:0005740 mitochondrial envelope
GO:0098800 inner mitochondrial membrane protein complex
C
GOterm
RichFactor
GeneNumber
250
500
750
1000
1250
qvalue
1.0e-49
7.5e-50
5.0e-50
2.5e-50
3.1e-82
D
Top 20 of KEGG Enrichment
Parkinson disease
Prion disease
Huntington disease
Oxidative phosphorylation
Chemical carcinogenesis - reactive oxygen species
Diabetic cardiomyopathy
Alzheimer disease
Thermogenesis
Ribosome
Amyotrophic lateral sclerosis
Pathways of neurodegeneration - multiple diseases
Non-alcoholic fatty liver disease
Proteasome
Retrograde endocannabinoid signaling
Spliceosome
Metabolic pathways
Adherens junction
Ribosome biogenesis in eukaryotes
Spinocerebellar ataxia
Cardiac muscle contraction
Pathway
RichFactor
GeneNumber
50
100
150
200
qvalue
1e-04
5e-05
B
Top 20 of GO Enrichment
GO:0003723 RNA binding
GO:0003735 structural constituent of ribosome
GO:0015453 oxidoreduction-driven active transmembrane transporter activity
GO:0009055 electron transfer activity
GO:0045296 cadherin binding
GO:0003955 NAD(P)H dehydrogenase (quinone) activity
GO:0050136 NADH dehydrogenase (quinone) activity
GO:0016655 oxidoreductase activity, acting on NAD(P)H, quinone or similar compound as acceptor
GO:0008137 NADH dehydrogenase (ubiquinone) activity
GO:0005515 protein binding
GO:0003954 NADH dehydrogenase activity
GO:0050839 cell adhesion molecule binding
GO:0003676 nucleic acid binding
GO:0016651 oxidoreductase activity, acting on NAD(P)H
GO:1901363 heterocyclic compound binding
GO:0097159 organic cyclic compound binding
GO:0016491 oxidoreductase activity
GO:0005198 structural molecule activity
GO:0015399 primary active transmembrane transporter activity
GO:0005488 binding
F
GOterm
RichFactor
GeneNumber
250
500
750
1000
1250
qvalue
7.5e-11
5.0e-11
2.5e-11
E
Top 20 of DO Enrichment
DOID:305 carcinoma
DOID:1749 squamous cell carcinoma
DOID:0050687 cell type cancer
DOID:3119 gastrointestinal system cancer
DOID:162 cancer
DOID:14566 disease of cellular proliferation
DOID:684 hepatocellular carcinoma
DOID:686 liver carcinoma
DOID:170 endocrine gland cancer
DOID:3571 liver cancer
DOID:3905 lung carcinoma
DOID:193 reproductive organ cancer
DOID:1612 breast cancer
DOID:5093 thoracic cancer
DOID:3459 breast carcinoma
DOID:0050686 organ system cancer
DOID:120 female reproductive organ cancer
DOID:2994 germ cell cancer
DOID:4766 embryoma
DOID:1324 lung cancer
DOterm
RichFactor
GeneNumber
100
200
300
400
500
qvalue
0.0020
0.0015
0.0010
0.0005
C
Top 20 of GO Enrichment
GO:1901566 organonitrogen compound biosynthetic process
GO:0009060 aerobic respiration
GO:0006119 oxidative phosphorylation
GO:0006412 translation
GO:0043043 peptide biosynthetic process
GO:0045333 cellular respiration
GO:0006518 peptide metabolic process
GO:0046034 ATP metabolic process
GO:0042773 ATP synthesis coupled electron transport
GO:0042775 mitochondrial ATP synthesis coupled electron transport
GO:0015985 energy coupled proton transport, down electrochemical gradient
GO:0015986 ATP synthesis coupled proton transport
GO:0043604 amide biosynthetic process
GO:0019646 aerobic electron transport chain
GO:0042776 mitochondrial ATP synthesis coupled proton transport
GO:0022904 respiratory electron transport chain
GO:0009145 purine nucleoside triphosphate biosynthetic process
GO:0015980 energy derivation by oxidation of organic compounds
GO:0009206 purine ribonucleoside triphosphate biosynthetic process
GO:0006091 generation of precursor metabolites and energy
P
GOterm
RichFactor
GeneNumber
50
100
150
200
250
300
qvalue
6.300e-31
4.725e-31
3.150e-31
1.575e-31
4.200e-44
F
Top 20 of reactome Enrichment
R-HSA-1428517 The citric acid (TCA) cycle and respiratory electron transport
R-HSA-163200 Respiratory electron transport, ATP synthesis by chemiosmotic coupling, and heat production by uncoupling proteins.
R-HSA-72766 Translation
R-HSA-611105 Respiratory electron transport
R-HSA-5389842 TUFM hydrolyzes GTP and TUFM:GDP dissociates from 55S ribosome
R-HSA-5389848 TUFM:GTP:aminoacyl-tRNA binds 55S ribosome:mRNA:fMet-tRNA
R-HSA-5389840 Mitochondrial translation elongation
R-HSA-5389857 Peptide transfer from P-site to A-site (peptide bond formation)
R-HSA-5419261 55S ribosome with peptidyl-tRNA in A site binds GFM1:GTP
R-HSA-5419264 MTRF1L (MTRF1a) or ICT1 binds stop codon in 55S ribosome:mRNA:peptidyl-tRNA
R-HSA-5419271 55S ribosome hydrolyzes peptidyl-tRNA bond and MTRF1L hydrolyzes GTP
R-HSA-5419279 Translocation of peptidyl-tRNA from A-site to P-site (and translocation of 55S ribosome by 3 bases along mRNA)
R-HSA-5419281 MRRF binds 55S ribosome:mRNA:tRNA
R-HSA-5389839 39S subunit binds 28S subunit:mRNA:fMet-tRNA
R-HSA-5419273 Hydrolysis of GTP and dissociation of 28S and 39S subunits
R-HSA-5419277 GFM2:GTP binds 55S ribosome:mRNA:tRNA:MRRF releasing mRNA and tRNA
R-HSA-5368286 Mitochondrial translation initiation
R-HSA-5419276 Mitochondrial translation termination
R-HSA-5368287 Mitochondrial translation
R-HSA-8953854 Metabolism of RNA
Reactome
RichFactor
GeneNumber
50
75
100
125
150
qvalue
1.80e-22
1.35e-22
9.00e-23
4.50e-23
9.10e-40

Supplementary 3. Comprehensive enrichment analyses of the upregulated gene set in cluster 1, containing GO, KEGG, DO and Reactome enrichments.

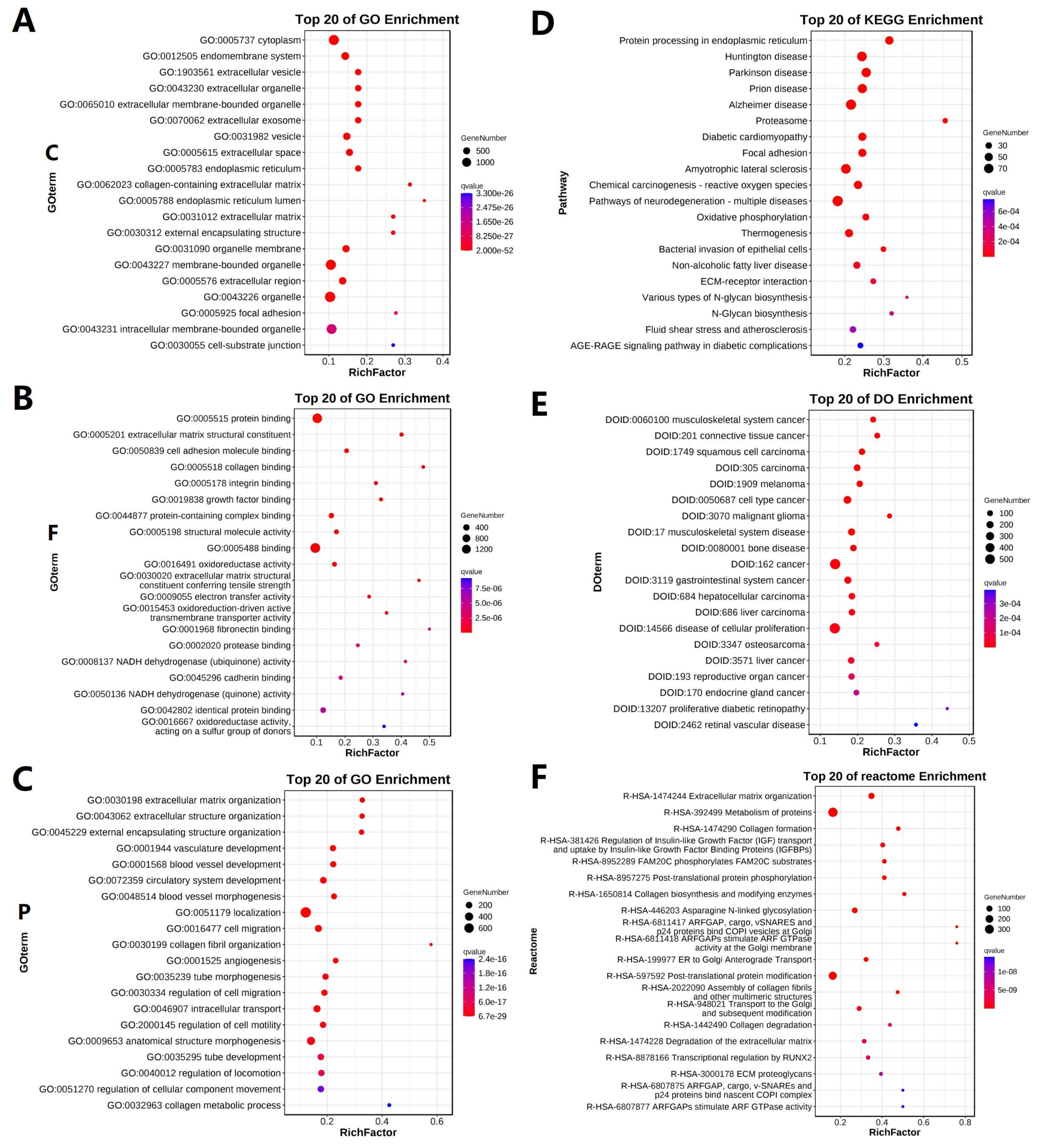
A
Top 20 of GO Enrichment
GO:0005737 cytoplasm
GO:0012505 endomembrane system
GO:1903561 extracellular vesicle
GO:0043230 extracellular organelle
GO:0065010 extracellular membrane-bounded organelle
GO:0070062 extracellular exosome
GO:0031982 vesicle
GO:0005615 extracellular space
GO:0005783 endoplasmic reticulum
GO:0062023 collagen-containing extracellular matrix
GO:0005788 endoplasmic reticulum lumen
GO:0031012 extracellular matrix
GO:0030312 external encapsulating structure
GO:0031090 organelle membrane
GO:0043227 membrane-bounded organelle
GO:0005576 extracellular region
GO:0043226 organelle
GO:0005925 focal adhesion
GO:0043231 intracellular membrane-bounded organelle
GO:0030055 cell-substrate junction
C
GOterm
RichFactor
0.1
0.2
0.3
0.4
GeneNumber
500
1000
qvalue
3.300e-26
2.475e-26
1.650e-26
8.250e-27
2.000e-52
D
Top 20 of KEGG Enrichment
Protein processing in endoplasmic reticulum
Huntington disease
Parkinson disease
Prion disease
Alzheimer disease
Proteasome
Diabetic cardiomyopathy
Focal adhesion
Amyotrophic lateral sclerosis
Chemical carcinogenesis - reactive oxygen species
Pathways of neurodegeneration - multiple diseases
Oxidative phosphorylation
Thermogenesis
Bacterial invasion of epithelial cells
Non-alcoholic fatty liver disease
ECM-receptor interaction
Various types of N-glycan biosynthesis
N-Glycan biosynthesis
Fluid shear stress and atherosclerosis
AGE-RAGE signaling pathway in diabetic complications
Pathway
RichFactor
0.2
0.3
0.4
0.5
GeneNumber
30
50
70
qvalue
6e-04
4e-04
2e-04
B
Top 20 of GO Enrichment
GO:0005515 protein binding
GO:0005201 extracellular matrix structural constituent
GO:0050839 cell adhesion molecule binding
GO:0005518 collagen binding
GO:0005178 integrin binding
GO:0019838 growth factor binding
GO:0044877 protein-containing complex binding
GO:0005198 structural molecule activity
GO:0005488 binding
GO:0016491 oxidoreductase activity
GO:0030020 extracellular matrix structural constituent conferring tensile strength
GO:0009055 electron transfer activity
GO:0015453 oxidoreduction-driven active transmembrane transporter activity
GO:0001968 fibronectin binding
GO:0002020 protease binding
GO:0008137 NADH dehydrogenase (ubiquinone) activity
GO:0045296 cadherin binding
GO:0050136 NADH dehydrogenase (quinone) activity
GO:0042802 identical protein binding
GO:0016667 oxidoreductase activity, acting on a sulfur group of donors
F
GOterm
RichFactor
0.1
0.2
0.3
0.4
0.5
GeneNumber
400
800
1200
qvalue
7.5e-06
5.0e-06
2.5e-06
E
Top 20 of DO Enrichment
DOID:0060100 musculoskeletal system cancer
DOID:201 connective tissue cancer
DOID:1749 squamous cell carcinoma
DOID:305 carcinoma
DOID:1909 melanoma
DOID:0050687 cell type cancer
DOID:3070 malignant glioma
DOID:17 musculoskeletal system disease
DOID:0080001 bone disease
DOID:162 cancer
DOID:3119 gastrointestinal system cancer
DOID:684 hepatocellular carcinoma
DOID:686 liver carcinoma
DOID:14566 disease of cellular proliferation
DOID:3347 osteosarcoma
DOID:3571 liver cancer
DOID:193 reproductive organ cancer
DOID:170 endocrine gland cancer
DOID:13207 proliferative diabetic retinopathy
DOID:2462 retinal vascular disease
DOterm
RichFactor
0.1
0.2
0.3
0.4
0.5
GeneNumber
100
200
300
400
500
qvalue
3e-04
2e-04
1e-04
C
Top 20 of GO Enrichment
GO:0030198 extracellular matrix organization
GO:0043062 extracellular structure organization
GO:0045229 external encapsulating structure organization
GO:0001944 vasculature development
GO:0001568 blood vessel development
GO:0072359 circulatory system development
GO:0048514 blood vessel morphogenesis
GO:0051179 localization
GO:0016477 cell migration
GO:0030199 collagen fibril organization
GO:0001525 angiogenesis
GO:0035239 tube morphogenesis
GO:0030334 regulation of cell migration
GO:0046907 intracellular transport
GO:2000145 regulation of cell motility
GO:0009653 anatomical structure morphogenesis
GO:0035295 tube development
GO:0040012 regulation of locomotion
GO:0051270 regulation of cellular component movement
GO:0032963 collagen metabolic process
P
GOterm
RichFactor
0.2
0.4
0.6
GeneNumber
200
400
600
qvalue
2.4e-16
1.8e-16
1.2e-16
6.0e-17
6.7e-29
F
Top 20 of reactome Enrichment
R-HSA-1474244 Extracellular matrix organization
R-HSA-392499 Metabolism of proteins
R-HSA-1474290 Collagen formation
R-HSA-381426 Regulation of Insulin-like Growth Factor (IGF) transport and uptake by Insulin-like Growth Factor Binding Proteins (IGFBPs)
R-HSA-8952289 FAM20C phosphorylates FAM20C substrates
R-HSA-8957275 Post-translational protein phosphorylation
R-HSA-1650814 Collagen biosynthesis and modifying enzymes
R-HSA-446203 Asparagine N-linked glycosylation
R-HSA-6811417 ARFGAP, cargo, vSNARES and p24 proteins bind COPI vesicles at Golgi
R-HSA-6811418 ARFGAPs stimulate ARF GTPase activity at the Golgi membrane
R-HSA-199977 ER to Golgi Anterograde Transport
R-HSA-597592 Post-translational protein modification
R-HSA-2022090 Assembly of collagen fibrils and other multimeric structures
R-HSA-948021 Transport to the Golgi and subsequent modification
R-HSA-1442490 Collagen degradation
R-HSA-1474228 Degradation of the extracellular matrix
R-HSA-8878166 Transcriptional regulation by RUNX2
R-HSA-3000178 ECM proteoglycans
R-HSA-6807875 ARFGAP, cargo, v-SNAREs and p24 proteins bind nascent COPI complex
R-HSA-6807877 ARFGAPs stimulate ARF GTPase activity
Reactome
RichFactor
0.2
0.4
0.6
0.8
GeneNumber
100
200
300
qvalue
1e-08
5e-09

Supplementary 4. Comprehensive enrichment analyses of the upregulated gene set in cluster 12, containing GO, KEGG, DO and Reactome enrichments.

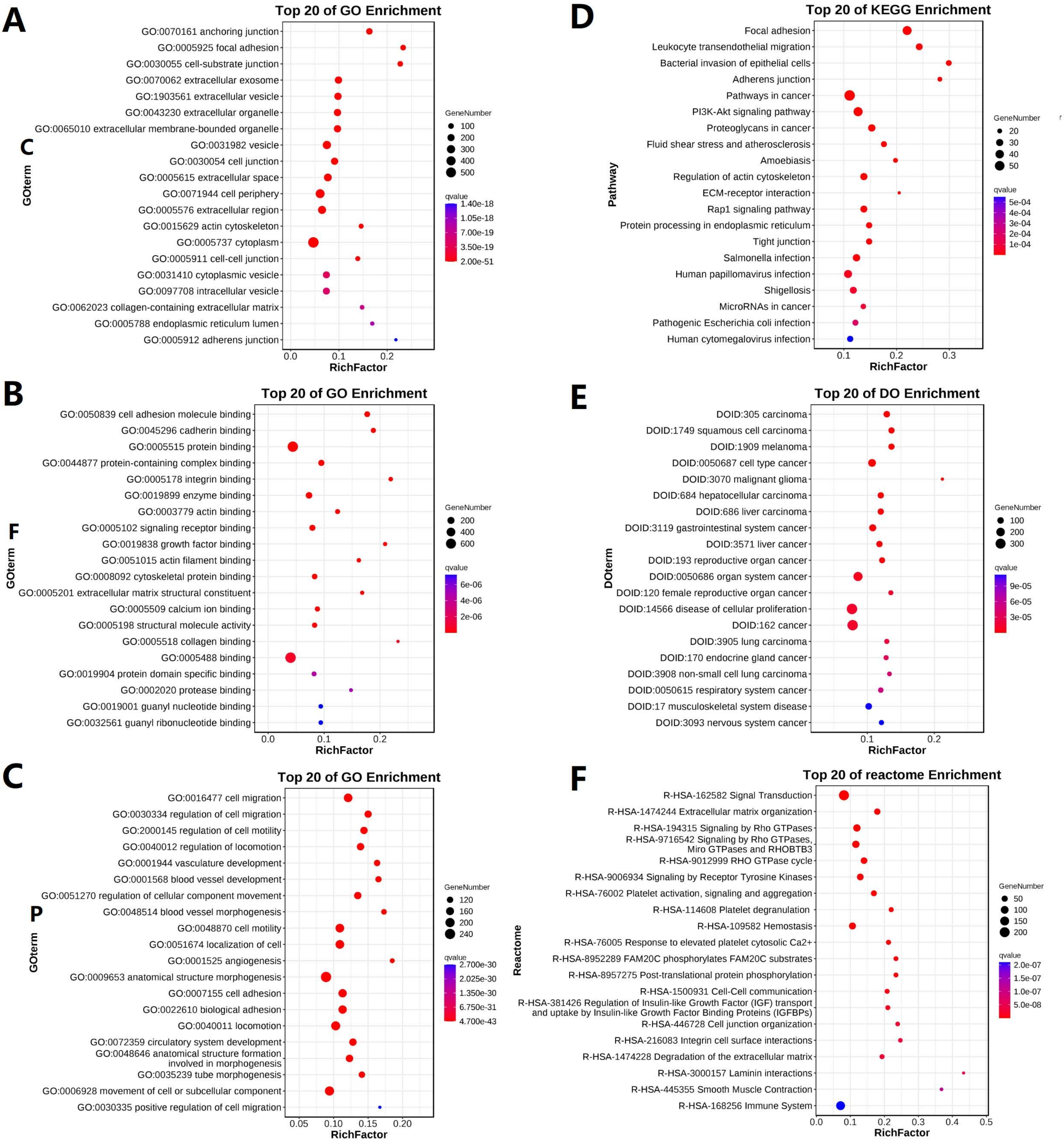

A
Top 20 of GO Enrichment
GO:0070161 anchoring junction
GO:0005925 focal adhesion
GO:0030055 cell-substrate junction
GO:0070062 extracellular exosome
GO:1903561 extracellular vesicle
GO:0043230 extracellular organelle
GO:0065010 extracellular membrane-bounded organelle
GO:0031982 vesicle
GO:0030054 cell junction
GO:0005615 extracellular space
GO:0071944 cell periphery
GO:0005576 extracellular region
GO:0015629 actin cytoskeleton
GO:0005737 cytoplasm
GO:0005911 cell-cell junction
GO:0031410 cytoplasmic vesicle
GO:0097708 intracellular vesicle
GO:0062023 collagen-containing extracellular matrix
GO:0005788 endoplasmic reticulum lumen
GO:0005912 adherens junction
GOterm
RichFactor
0.0
0.1
0.2
GeneNumber
100
200
300
400
500
qvalue
1.40e-18
1.05e-18
7.00e-19
3.50e-19
2.00e-51
D
Top 20 of KEGG Enrichment
Focal adhesion
Leukocyte transendothelial migration
Bacterial invasion of epithelial cells
Adherens junction
Pathways in cancer
PI3K-Akt signaling pathway
Proteoglycans in cancer
Fluid shear stress and atherosclerosis
Amoebiasis
Regulation of actin cytoskeleton
ECM-receptor interaction
Rap1 signaling pathway
Protein processing in endoplasmic reticulum
Tight junction
Salmonella infection
Human papillomavirus infection
Shigellosis
MicroRNAs in cancer
Pathogenic Escherichia coli infection
Human cytomegalovirus infection
Pathway
RichFactor
0.1
0.2
0.3
GeneNumber
20
30
40
50
qvalue
5e-04
4e-04
3e-04
2e-04
1e-04
B
Top 20 of GO Enrichment
GO:0050839 cell adhesion molecule binding
GO:0045296 cadherin binding
GO:0005515 protein binding
GO:0044877 protein-containing complex binding
GO:0005178 integrin binding
GO:0019899 enzyme binding
GO:0003779 actin binding
GO:0005102 signaling receptor binding
GO:0019838 growth factor binding
GO:0051015 actin filament binding
GO:0008092 cytoskeletal protein binding
GO:0005201 extracellular matrix structural constituent
GO:0005509 calcium ion binding
GO:0005198 structural molecule activity
GO:0005518 collagen binding
GO:0005488 binding
GO:0019904 protein domain specific binding
GO:0002020 protease binding
GO:0019001 guanyl nucleotide binding
GO:0032561 guanyl ribonucleotide binding
GOterm
RichFactor
0.0
0.1
0.2
GeneNumber
200
400
600
qvalue
6e-06
4e-06
2e-06
E
Top 20 of DO Enrichment
DOID:305 carcinoma
DOID:1749 squamous cell carcinoma
DOID:1909 melanoma
DOID:0050687 cell type cancer
DOID:3070 malignant glioma
DOID:684 hepatocellular carcinoma
DOID:686 liver carcinoma
DOID:3119 gastrointestinal system cancer
DOID:3571 liver cancer
DOID:193 reproductive organ cancer
DOID:0050686 organ system cancer
DOID:120 female reproductive organ cancer
DOID:14566 disease of cellular proliferation
DOID:162 cancer
DOID:3905 lung carcinoma
DOID:170 endocrine gland cancer
DOID:3908 non-small cell lung carcinoma
DOID:0050615 respiratory system cancer
DOID:17 musculoskeletal system disease
DOID:3093 nervous system cancer
DOterm
RichFactor
0.1
0.2
GeneNumber
100
200
300
qvalue
9e-05
6e-05
3e-05
C
Top 20 of GO Enrichment
GO:0016477 cell migration
GO:0030334 regulation of cell migration
GO:2000145 regulation of cell motility
GO:0040012 regulation of locomotion
GO:0001944 vasculature development
GO:0001568 blood vessel development
GO:0051270 regulation of cellular component movement
GO:0048514 blood vessel morphogenesis
GO:0048870 cell motility
GO:0051674 localization of cell
GO:0001525 angiogenesis
GO:0009653 anatomical structure morphogenesis
GO:0007155 cell adhesion
GO:0022610 biological adhesion
GO:0040011 locomotion
GO:0072359 circulatory system development
GO:0048646 anatomical structure formation involved in morphogenesis
GO:0035239 tube morphogenesis
GO:0006928 movement of cell or subcellular component
GO:0030335 positive regulation of cell migration
GOterm
RichFactor
0.05
0.10
0.15
0.20
GeneNumber
120
160
200
240
qvalue
2.700e-30
2.025e-30
1.350e-30
6.750e-31
4.700e-43
F
Top 20 of reactome Enrichment
R-HSA-162582 Signal Transduction
R-HSA-1474244 Extracellular matrix organization
R-HSA-194315 Signaling by Rho GTPases
R-HSA-9716542 Signaling by Rho GTPases, Miro GTPases and RHOBTB3
R-HSA-9012999 RHO GTPase cycle
R-HSA-9006934 Signaling by Receptor Tyrosine Kinases
R-HSA-76002 Platelet activation, signaling and aggregation
R-HSA-114608 Platelet degranulation
R-HSA-109582 Hemostasis
R-HSA-76005 Response to elevated platelet cytosolic Ca2+
R-HSA-8952289 FAM20C phosphorylates FAM20C substrates
R-HSA-8957275 Post-translational protein phosphorylation
R-HSA-1500931 Cell-Cell communication
R-HSA-381426 Regulation of Insulin-like Growth Factor (IGF) transport and uptake by Insulin-like Growth Factor Binding Proteins (IGFBPs)
R-HSA-446728 Cell junction organization
R-HSA-216083 Integrin cell surface interactions
R-HSA-1474228 Degradation of the extracellular matrix
R-HSA-3000157 Laminin interactions
R-HSA-445355 Smooth Muscle Contraction
R-HSA-168256 Immune System
Reactome
RichFactor
0.0
0.1
0.2
0.3
0.4
0.5
GeneNumber
50
100
150
200
qvalue
2.0e-07
1.5e-07
1.0e-07
5.0e-08

Supplementary 5. Comprehensive enrichment analyses of the upregulated gene set in cluster 14, containing GO, KEGG, DO and Reactome enrichments.
